# Supplementary material for: Mitochondrial Fis1 tunes Rab7A activity via TBC1D15 modulating STING degradation
Source: Cell Rep. Author manuscript; Available in PMC 2026 Oct 14. (PMC13667912; doi:10.1016/j.celrep.2026.117994)
Supplement: Supplementary file 1 [file NIHMS2212243-supplement-1.pdf]

Cell Reports, Volume *45*

Supplemental information

Mitochondrial Fis1 tunes Rab7A activity via TBC1D15 modulating STING degradation

Eunice Dominguez-Martin, Linlin Lei, Jennifer D. Petersen, Peng-Peng Zhu, Katherine R. Tucker, and Richard J. Youle

Figure S1. STING degradation is impaired in the absence of Fis1, related to Figure 1. **(A)** STING, Fis1 and Hsp90 protein levels detected by immunoblotting whole-cell lysates from WT and *FIS1*$^{ko\#10}$ cells treated with cGAMP or left untreated. **(B)** Densitometric quantifications of STING levels in experiments as in A (NT= no treatment, AU= Arbitrary units, n=3, mean±SD, paired t test: WT+cGAMP vs *FIS1*$^{ko\#10}$+cGAMP). **(C)** Immunoblot to detect STING and Fis1 protein levels in whole-cell lysates from WT, *FIS1*$^{ko\#13}$, WT+pBABEpuro or *FIS1*$^{ko\#13}$+pBABEpuro, induced with cGAMP or left untreated. Actin was included as a loading control. **(D)** Quantification of STING protein levels of experiments as in C (AU= Arbitrary units, n=3, mean±SD, one-way ANOVA, Bonferroni). **(E)** STING degradation in WT and *FIS1*$^{ko\#}$ HCT116 cells treated with cGAMP or left untreated. Actin was used as loading control and Fis1 absence was corroborated. **(F)** STING protein levels as a ratio of cGAMP/NT of experiments as in (E) (n=3, mean±SD, two-tailed paired *t-test*). **(G)** Mitophagy induction determined by PINK1 stabilization and detected by immunoblotting whole cell lysates of WT HeLa cells stably expressing mCherry-Parkin (mCh-PRKN) after the specified treatments. STING was also blotted, and Actin was included as a loading control. **(H)** Immunoblot assaying STING levels in WT, *FIS1*$^{ko\#13}$, *FIS1*$^{ko\#13}$+Fis1$^{\Delta C}$ and *FIS1*$^{ko\#13}$+Fis1^{WT} cells stimulated with cGAMP or with no treatment (NT). Fis1 expression was verified, and Actin was blotted as a loading control. **(I)** STING protein levels quantification as a ratio of cGAMP/NT of experiments in (H) (NT= no treatment, AU= Arbitrary units, n=3, mean±SD, one-way ANOVA, Bonferroni). **(J)** STING protein levels assayed by immunoblot in *FIS1*$^{ko\#13}$ cells stably expressing either Fis1^{WT}, a cytosolic C-term truncation: Fis1$^{\Delta C}$, or the Fis1$^{\Delta C\text{-}OMP25}$ mitochondria-anchored chimera. Fis1 was also detected, and Actin was blotted as a loading control. **(K)** Densitometries of STING protein levels as a ratio of cGAMP/NT of experiments as in (H) (n=3, mean±SD, one-way ANOVA, Bonferroni). **(L)** Confocal images of *FIS1*$^{ko\#13}$ cells expressing either Fis1^{WT}, Fis1$^{\Delta C}$, or Fis1$^{\Delta C\text{-}OMP25}$ processed for the detection of Fis1 and TOMM20 by immunofluorescence. The Pearson's correlation of Fis1 vs TOMM20 was quantified. Scale bar = 10 µm. **(M-N)** Plots of the natural logarithm (ln) of STING **(M)** or TIMM17A **(N)** normalized protein levels relative to t=0h from 3 experiments as in Figure 1D. A linear regression was obtained from the data plotted in Figure 1 and the respective line equations are displayed.

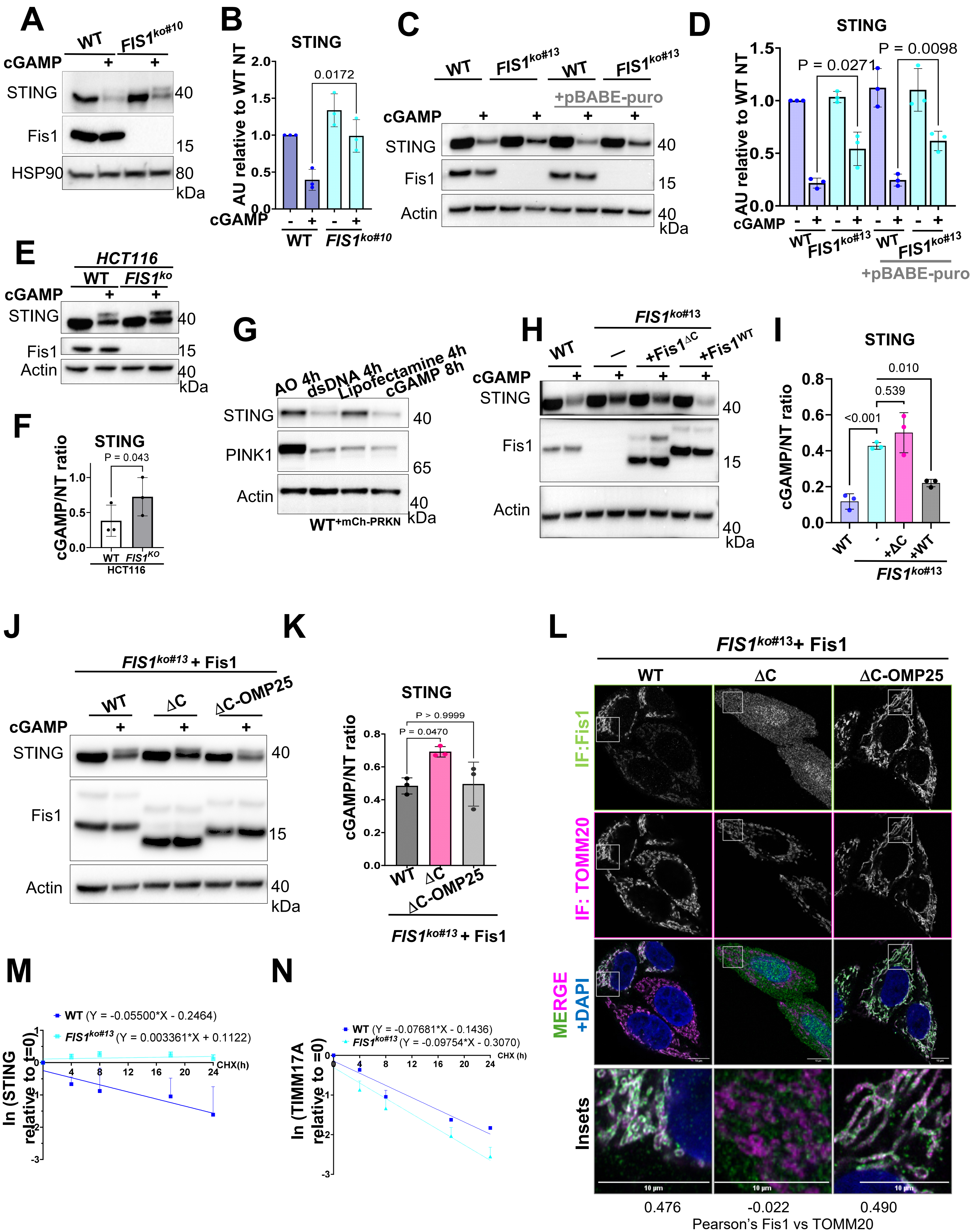

A
WT
FIS1ko#10
cGAMP
STING
Fis1
HSP90
40
15
80
kDa
B
STING
AU relative to WT NT
0.0172
cGAMP
WT
FIS1ko#10
C
WT
FIS1ko#13
+pBABE-puro
cGAMP
STING
Fis1
Actin
D
STING
P = 0.0271
P = 0.0098
AU relative to WT NT
cGAMP
+pBABE-puro
E
HCT116
WT
FIS1ko
cGAMP
STING
Fis1
Actin
F
STING
P = 0.043
cGAMP/NT ratio
HCT116
G
AO 4h
dsDNA 4h
Lipofectamine 4h
cGAMP 8h
STING
PINK1
Actin
WT+mCh-PRKN
H
FIS1ko#13
WT
+Fis1ΔC
+Fis1WT
cGAMP
STING
Fis1
Actin
I
STING
cGAMP/NT ratio
<0.001
0.539
0.010
J
FIS1ko#13 + Fis1
WT
ΔC
ΔC-OMP25
cGAMP
STING
Fis1
Actin
K
STING
P > 0.9999
P = 0.0470
cGAMP/NT ratio
FIS1ko#13 + Fis1
L
FIS1ko#13+ Fis1
WT
ΔC
ΔC-OMP25
IF:Fis1
IF: TOMM20
MERGE
+DAPI
Insets
10 μm
0.476
-0.022
0.490
Pearson's Fis1 vs TOMM20
M
WT (Y = -0.05500*X - 0.2464)
FIS1ko#13 (Y = 0.003361*X + 0.1122)
ln (STING relative to t=0)
CHX(h)
N
WT (Y = -0.07681*X - 0.1436)
FIS1ko#13 (Y = -0.09754*X - 0.3070)
ln (TIMM17A relative to t=0)
CHX (h)

Figure S2. STING trafficking in WT and *FIS1*^{ko} HeLa cells, related to Figure 2.

Confocal microscopy analysis of STING subcellular localization in WT and *FIS1*$^{ko\#13}$ HeLa cells expressing mEGFP-STING. Cells were treated with cGAMP for the indicated times (h) and stained to detect by immunofluorescence the indicated marker proteins: PDI (Endoplasmic Reticulum), TGN46 (Trans Golgi Network). Scale bars: 10µm.

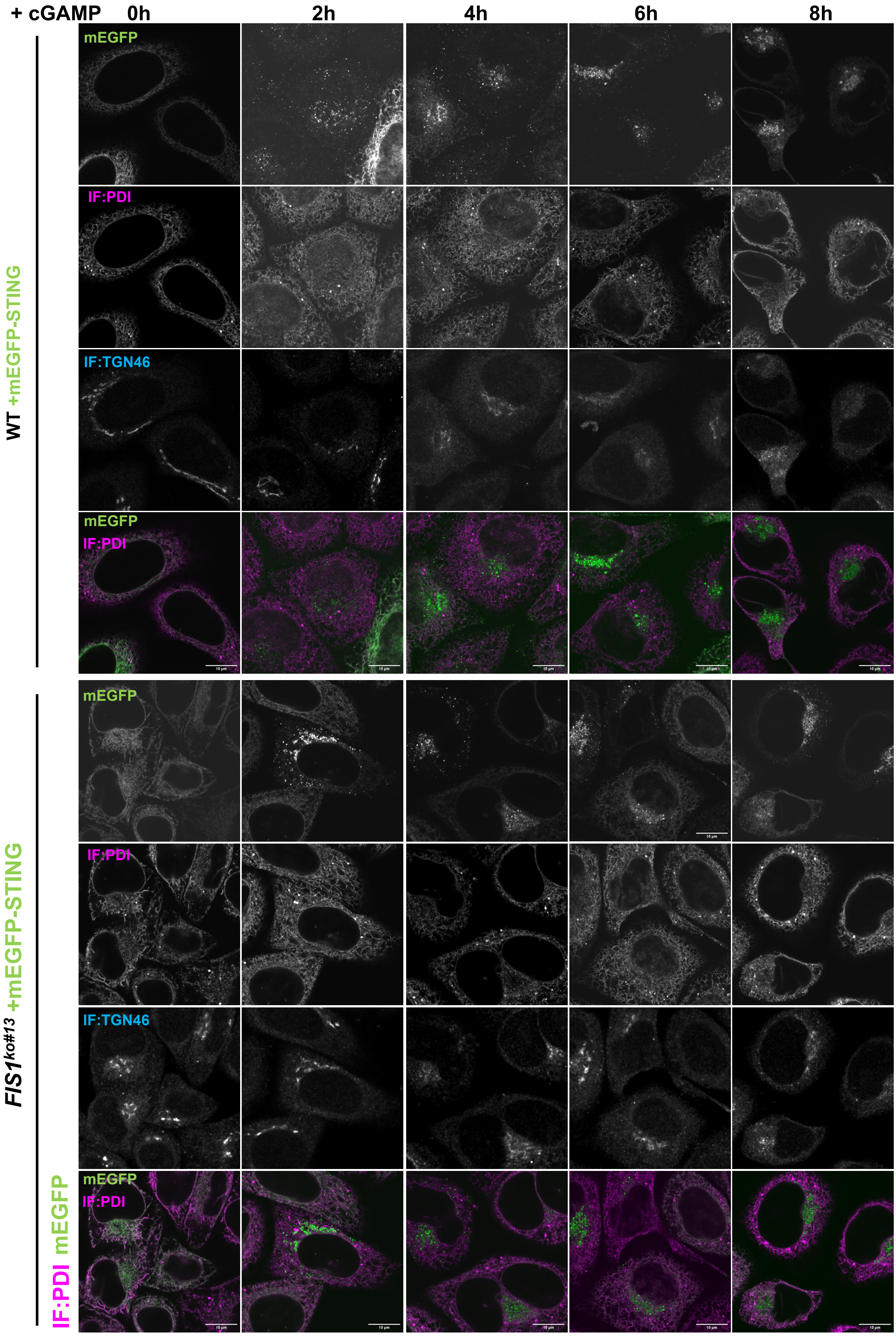

+ cGAMP
0h
2h
4h
6h
8h
WT +mEGFP-STING
mEGFP
IF:PDI
IF:TGN46
mEGFP
IF:PDI
FIS1ko#13 +mEGFP-STING
mEGFP
IF:PDI
IF:TGN46
mEGFP
IF:PDI
IF:PDI mEGFP

Figure S3. Rab7A localization during STING trafficking, related to Figure 3.
(A) Confocal microscopy time-course imaging of mCherry-Rab7 localization in WT or *FIS1*$^{ko\#13}$ cells expressing mEGFP-STING, treated with cGAMP for the indicated times (h) and stained to detect by immunofluorescence TGN46 (Trans Golgi Network 46). Scale bars: 10µm. **(B)** Immunoblot analysis of STING in WT and *RAB5*^{kd} cells upon a cGAMP treatment. Rab5 expression was assessed and HSP90 was used as loading control. **(C)** Densitometric quantification of immunoblot signals from (B) (n=3, mean ±SD, paired two-tailed *t-test*: WT vs *RAB5*^{kd}). **(D)** STING protein levels in WT and *RAB11*^{kd} cells upon a cGAMP treatment. Rab11 expression was assessed and HSP90 was used as loading control. **(C)** Densitometric quantification of immunoblot signals from (D) (n=3, mean ±SD, paired two-tailed *t-test*: WT vs *RAB11*^{kd}).

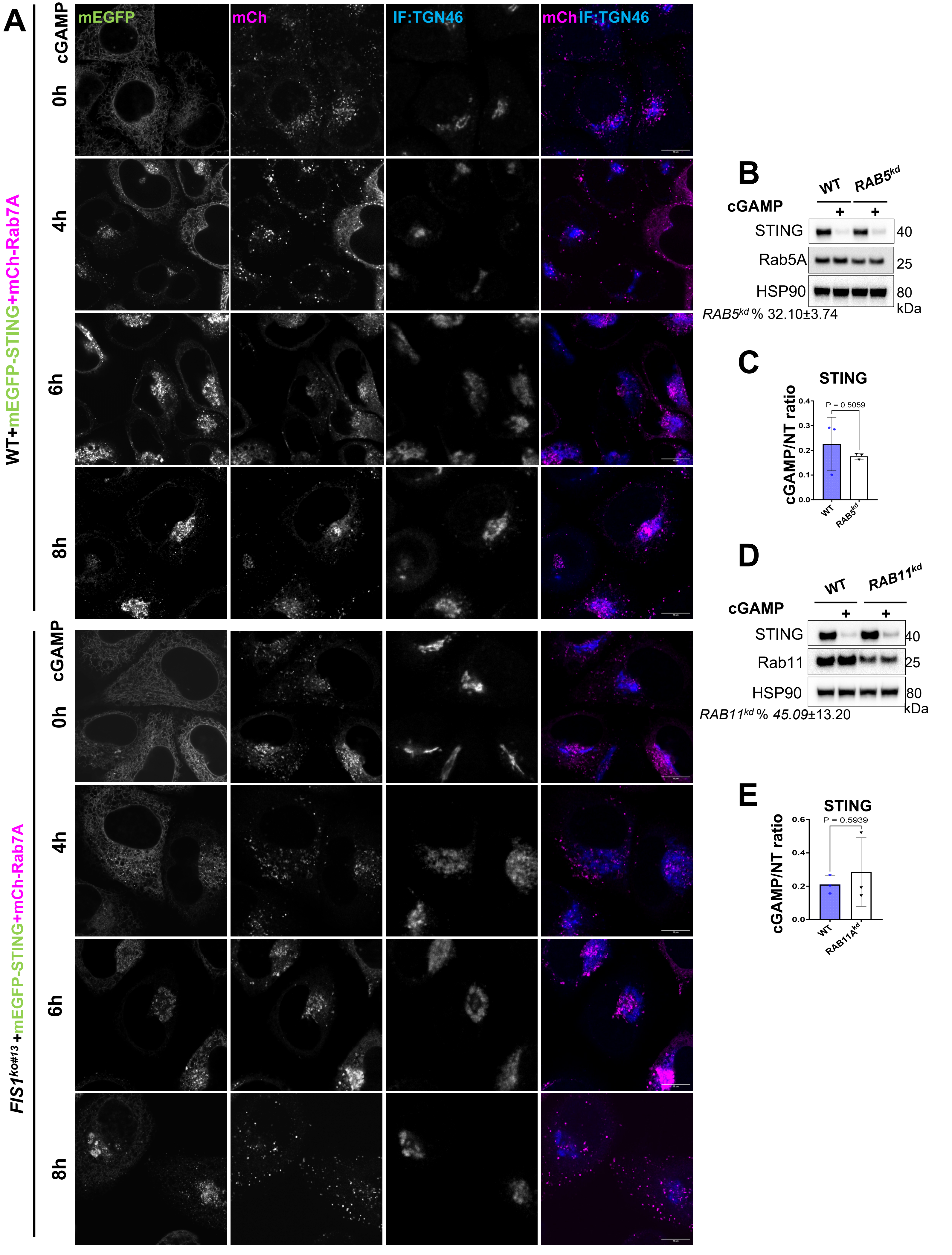
A
mEGFP
mCh
IF:TGN46
mCh IF:TGN46
cGAMP
0h
4h
6h
8h
WT+mEGFP-STING+mCh-Rab7A
FIS1ko#13+mEGFP-STING+mCh-Rab7A
B
WT
RAB5kd
cGAMP
+
+
STING
Rab5A
HSP90
40
25
80
kDa
RAB5kd % 32.10±3.74
C
STING
P = 0.5059
cGAMP/NT ratio
0.4
0.3
0.2
0.1
0.0
WT
RAB5kd
D
WT
RAB11kd
cGAMP
+
+
STING
Rab11
HSP90
40
25
80
kDa
RAB11kd % 45.09±13.20
E
STING
P = 0.5939
cGAMP/NT ratio
0.6
0.4
0.2
0.0
WT
RAB11Akd

Figure S4. The absence of Fis1 or TBC1D15 does not alter mitochondrial morphology and does not induce cGAMP accumulation in HeLa cells, related to Figure 4. (A) STING and TBC1D15 protein levels assayed in WT or *TBC1D15$^{ko\#8}$* cells upon cGAMP stimulation. Actin was blotted as a loading control. **(B)** Densitometries of STING levels in A (NT= no treatment, AU= Arbitrary units,n=3, mean±SD, paired t test: WT+cGAMP vs *TBC1D15$^{ko\#8}$*+cGAMP). **(C)** Immunoblot to detect STING levels in WT and *TBC1D15$^{ko\#5}$* cells with and without the expression of pHAGE-mEGFP upon a cGAMP treatment. **(D)** Quantification of STING protein levels as a ratio of cGAMP/NT of experiments as in C (n=3, mean±SD, one-way ANOVA, Bonferroni). **(E to J)** Representative maximum projections of Airy-scan processed confocal images in which mitochondria were labeled by immunofluorescence against TOMM20 in WT **(E)**, *DRP1^{ko}* **(F)**, *FIS1$^{ko\#13}$* **(G)**, *FIS1$^{ko\#10}$***(H)**, *TBC1D15$^{ko\#5}$* **(I)** or *TBC1D15$^{ko\#8}$* **(J)** cells. Nuclei were stained with DAPI. Insets show magnifications of peripheral mitochondria. Scale bar = 10 µm. **(K)** cGAMP levels (pg/µg of protein) detected by ELISA in whole cell lysates of WT, *FIS1$^{ko\#13}$* or *TBC1D15$^{ko\#5}$* cells stably expressing mCh-PRKN after an Antimycin + Oligomycin (AO) or mock treatment for 4h, a lysate of WT cells transfected with 4 µg/ml of poly-dAdT (dsDNA) was included as a positive control (dsDNA) (one-way ANOVA, Bonferrioni). For all images, *TBC1D15^{ko}=D15^{ko}*.

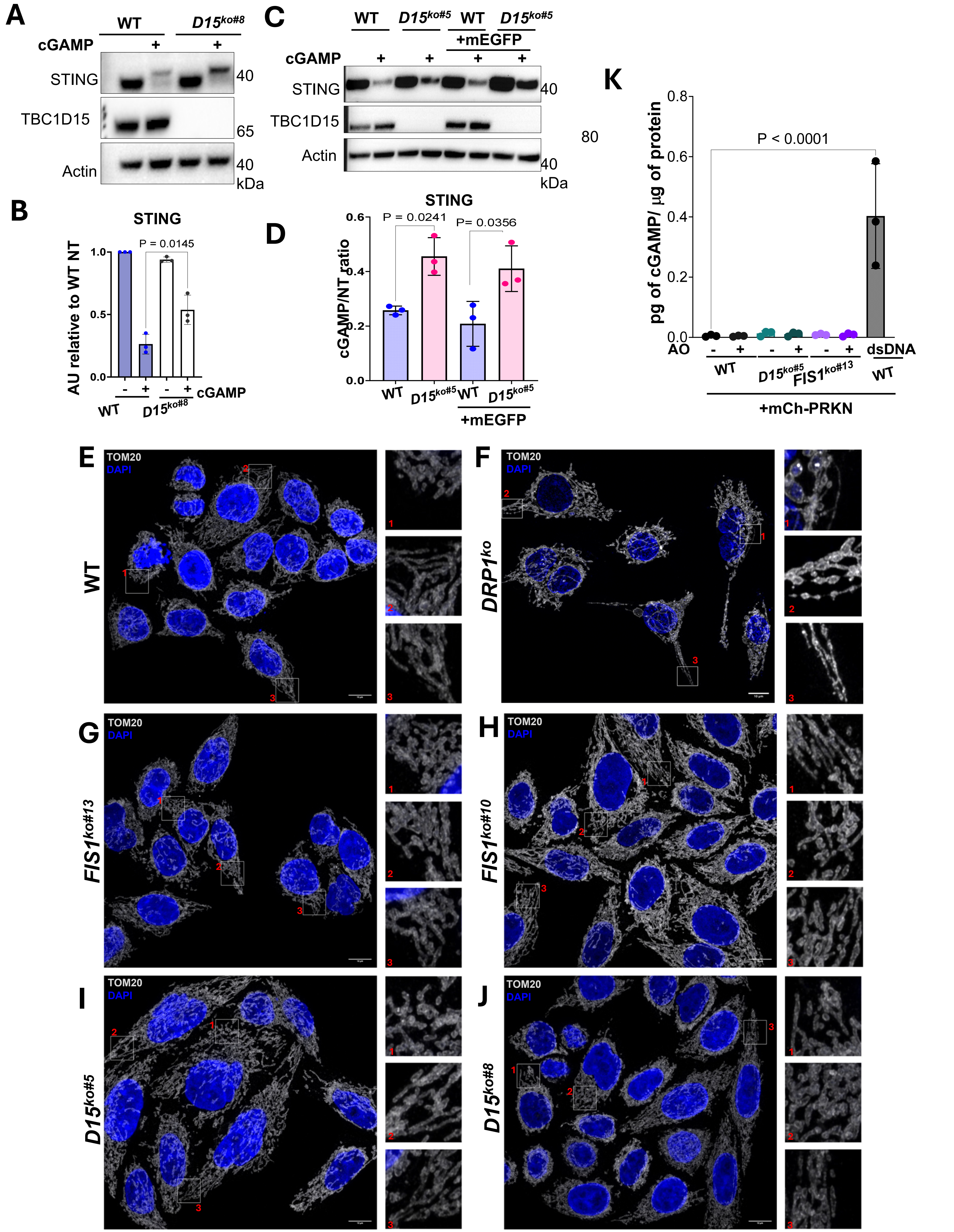

A
WT
D15ko#8
cGAMP
STING
TBC1D15
Actin
40
65
40
kDa
B
STING
AU relative to WT NT
P = 0.0145
cGAMP
WT
D15ko#8
C
WT
D15ko#5
WT
D15ko#5
+mEGFP
cGAMP
STING
TBC1D15
Actin
40
80
40
kDa
D
STING
cGAMP/NT ratio
P = 0.0241
P= 0.0356
WT
D15ko#5
WT
D15ko#5
+mEGFP
K
pg of cGAMP/ µg of protein
P < 0.0001
AO
dsDNA
WT
D15ko#5
FIS1ko#13
WT
+mCh-PRKN
E
WT
TOM20
DAPI
F
DRP1ko
G
FIS1ko#13
H
FIS1ko#10
I
D15ko#5
J
D15ko#8

Figure S5. mEGFP-TBC1D15 subcellular localization is influenced by Fis1 expression levels, relative to Figures 5 and 6. (A) mEGFP-TBC1D15 subcellular localization in *FIS1/TBC1D15*$^{dko\#9}$ cells lacking Fis1 expression (-) or in which either Fis1^{WT} or cytosolic Fis1$^{\Delta C}$ was reintroduced. Fis1 was detected via immunofluorescence. Images are representative stitched Airy-scan processed tiles. **(B)** Pearson's correlation was measured to assess colocalization of mEGFP-TBC1D15 and Fis1 forms in (A) (n=17 and 18 cells, two-tailed unpaired *t-test*). **(C)** Colocalization of mEGFP-TBC1D15 with TOMM20 detected by immunofluorescence acquired by imaging *FIS1/TBC1D15*$^{dko\#9}$ cells stably expressing mSca-STING and both Fis1 and mEGFP-TBC1D15. **(D)** Airy-scan processed images of mEGFP-TBC1D15 expressed in *FIS1/TBC1D15*$^{dko\#9}$+mSca-STING cells. The TGN was detected by staining TGN46 by immunofluorescence. **(E)** Subcellular localization of mCh-TBC1D15 in *FIS1/TBC1D15*$^{dko\#9}$ cells expressing either mEGFP-tagged WT Fis1 or ∆77-98 mutant Fis1. Mitochondria were detected by immunostaining TOMM20 and the TGN by detecting TGN46. **(F)** Pearson's coefficient of mCh-TBC1D15 vs TOMM20 in cells expressing either of WT Fis1 or ∆77-98 mutant Fis1 was quantified to assess mitochondria localization of TBC1D15 (n= 9 and 8, unpaired two-tailed *t-test*) and of **(G)** mCh-TBC1D15 vs TGN46 to assess TBC1D15 localization at Golgi (n= 7 and 10, unpaired two-tailed *t-test*). In all images, the scale bar = 10µm. For all images, *TBC1D15=D15*.

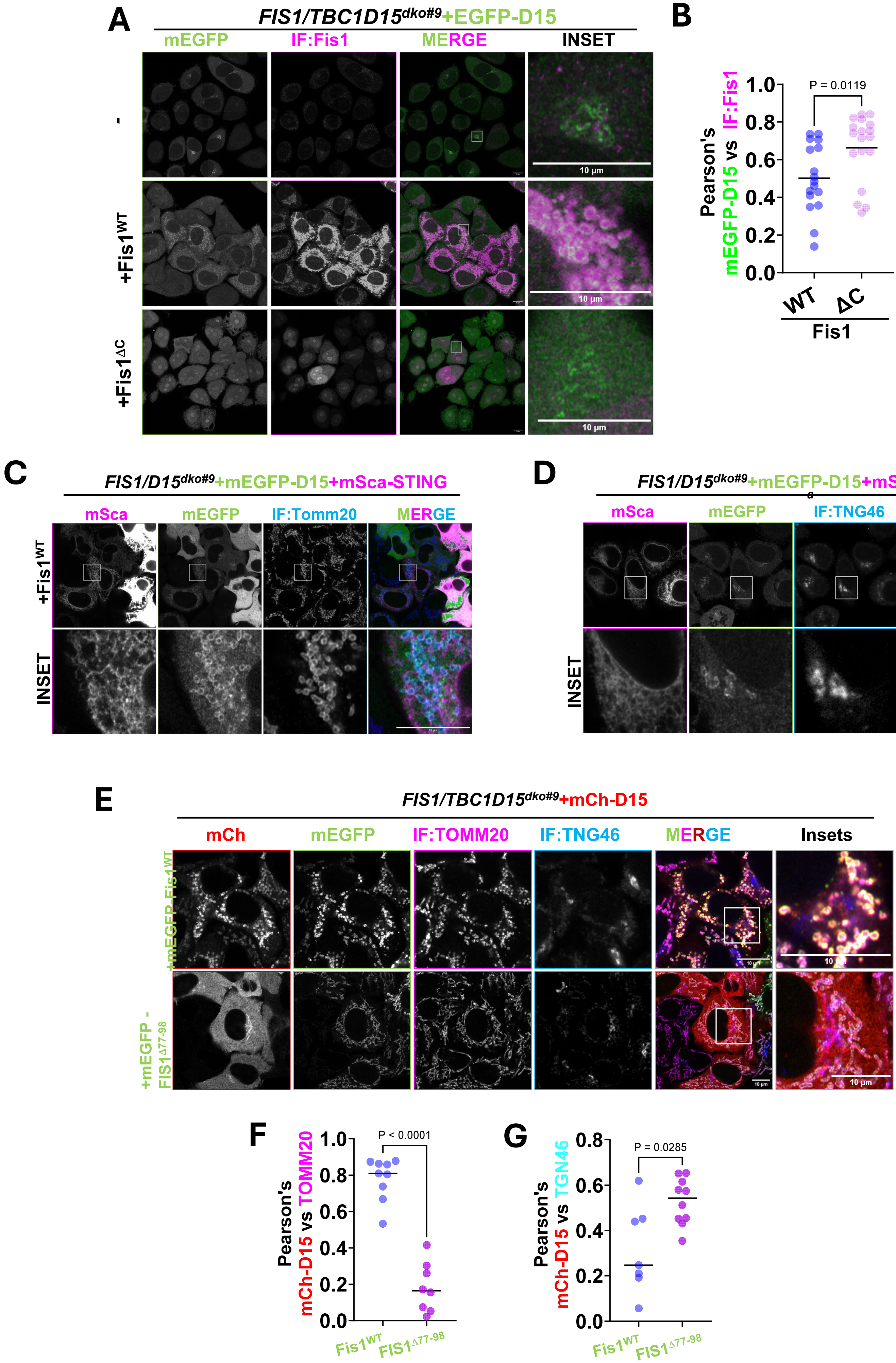
A
FIS1/TBC1D15dko#9+EGFP-D15
mEGFP
IF:Fis1
MERGE
INSET
-
+Fis1WT
+Fis1ΔC
10 µm
B
P = 0.0119
Pearson's mEGFP-D15 vs IF:Fis1
1.0
0.8
0.6
0.4
0.2
0.0
WT
ΔC
Fis1
C
FIS1/D15dko#9+mEGFP-D15+mSca-STING
mSca
mEGFP
IF:Tomm20
MERGE
+Fis1WT
INSET
D
FIS1/D15dko#9+mEGFP-D15+mSca-STING
mSca
mEGFP
IF:TNG46
MERGE
INSET
E
FIS1/TBC1D15dko#9+mCh-D15
mCh
mEGFP
IF:TOMM20
IF:TNG46
MERGE
Insets
+mEGFP-Fis1WT
+mEGFP - FIS1Δ77-98
10 µm
F
P < 0.0001
Pearson's mCh-D15 vs TOMM20
Fis1WT
FIS1Δ77-98
G
P = 0.0285
Pearson's mCh-D15 vs TGN46
Fis1WT
FIS1Δ77-98
