# Supplementary material for: Mitochondrial Fis1 tunes Rab7A activity via TBC1D15 modulating STING degradation
Source: Cell Rep. Author manuscript; Available in PMC 2026 Oct 14. (PMC13667912; doi:10.1016/j.celrep.2026.117994)
Supplement: Supplementary file 2 [file NIHMS2212243-supplement-2.pdf]

Cell Reports

Article

Mitochondrial Fis1 tunes Rab7A activity via TBC1D15 modulating STING degradation

Graphical abstract

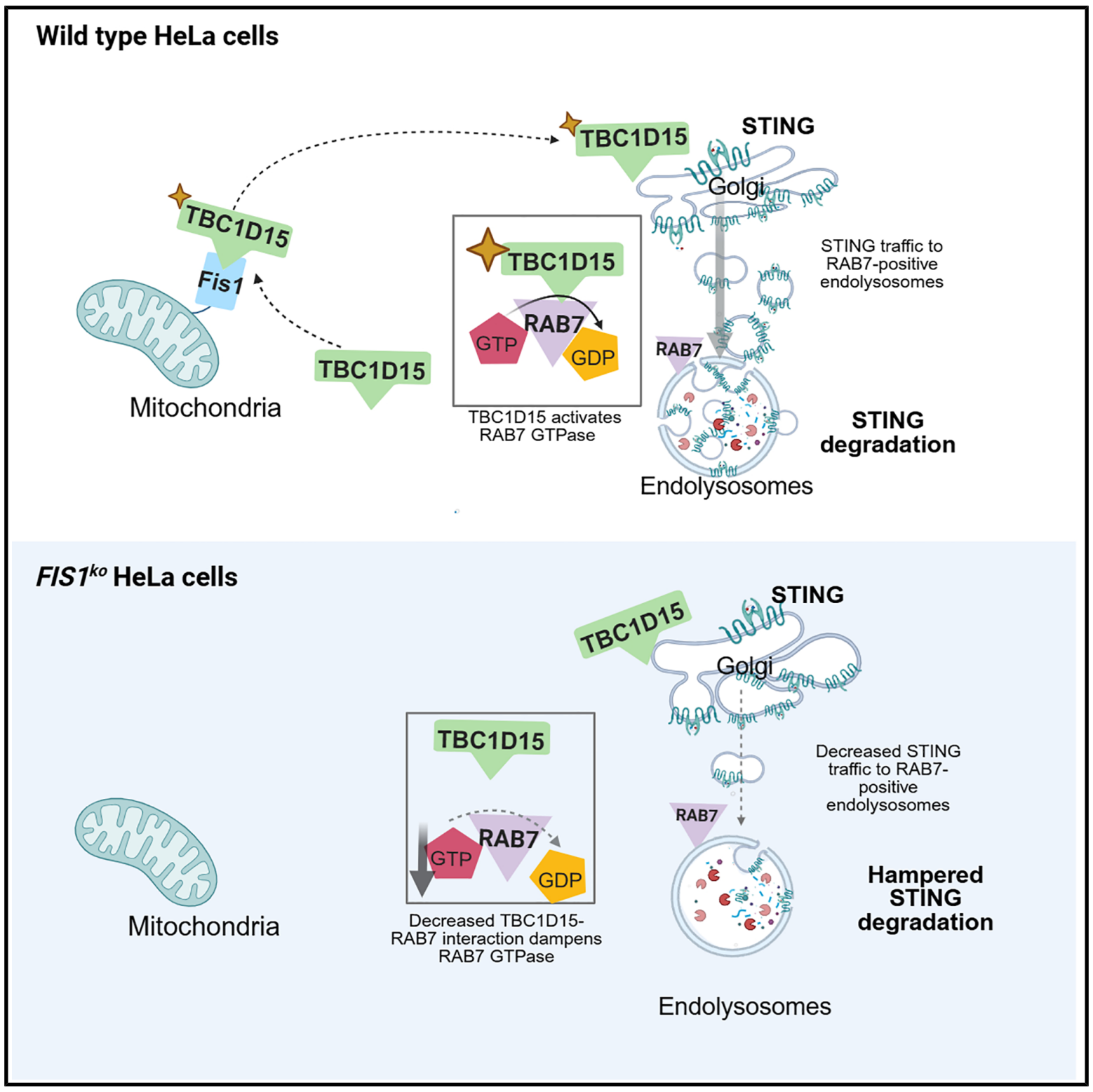

Highlights

- Loss of mitochondrial Fis1 hinders STING degradation
- Recruitment of the Rab-GAP TBC1D15 to mitochondria by Fis1 increases its affinity for Rab7A
- TBC1D15 localization shifts from mitochondria to the Golgi upon STING activation

Authors

Eunice Dominguez-Martin, Linlin Lei, Jennifer D. Petersen, Peng-Peng Zhu, Katherine R. Tucker, Richard J. Youle

Correspondence

eunice.dominguezmartin@jefferson.edu (E.D.-M.),
youler@ninds.nih.gov (R.J.Y.)

In brief

Dominguez-Martin et al. reveal a link between mitochondria and STING degradation. Fis1 recruits the Rab-GAP TBC1D15 to the mitochondria increasing its affinity for Rab7A. Upon STING activation, TBC1D15 translocates to the Golgi. The absence of the Fis1-TBC1D15 axis hampers STING late-stage trafficking and degradation.

Dominguez-Martin et al., 2026, Cell Reports *45*, 117994
September 22, 2026 Published by Elsevier Inc.
https://doi.org/10.1016/j.celrep.2026.117994

CellPress

Cell Reports

CellPress
OPEN ACCESS

Article

Mitochondrial Fis1 tunes Rab7A activity via TBC1D15 modulating STING degradation

Eunice Dominguez-Martin,[1,3,*] Linlin Lei,[1] Jennifer D. Petersen,[1,2] Peng-Peng Zhu,[1] Katherine R. Tucker,[1] and Richard J. Youle[1,4,*]

[1]Surgical Neurology Branch, National Institute of Neurological Disorders and Stroke, National Institutes of Health (NINDS), Bethesda, MD 20892, USA

[2]Electron Microscopy Core Facility, National Institute of Neurological Disorders and Stroke, National Institutes of Health (NINDS), Bethesda, MD 20892, USA

[3]Present address: MitoCare Center, Department of Pathology and Genomic Medicine, Thomas Jefferson University, Philadelphia, PA 19107, USA

[4]Lead contact

*Correspondence: eunice.dominguezmartin@jefferson.edu (E.D.-M.), youler@ninds.nih.gov (R.J.Y.)

https://doi.org/10.1016/j.celrep.2026.117994

SUMMARY

The mechanisms governing the final trafficking steps of the cyclic dinucleotide innate immune receptor stimulator of interferon genes (STING) are not fully understood. Here, we identify the mitochondrial protein Fis1 as a regulator of STING endolysosomal degradation. The absence of Fis1 in HeLa cells stabilizes STING, boosting downstream signaling without affecting its initial endoplasmic reticulum (ER)-to-Golgi traffic. Instead, Fis1 loss impairs STING delivery to endolysosomes. Fis1 recruits the Rab7A-GAP TBC1D15 to mitochondria and upon STING activation, TBC1D15 localization shifts to the Golgi. In the absence of Fis1, the interaction between TBC1D15 and Rab7A is disrupted, leading to decreased Rab7A GTPase activity and impaired STING degradation. Our findings reveal a mitochondria-controlled axis where Fis1 tunes Rab7A activity via TBC1D15, which is required to ensure proper STING degradation, directly linking mitochondria to lysosomal trafficking and the termination of innate immune signaling.

INTRODUCTION

Fis1 is a C-tail-anchored outer mitochondrial membrane protein with a cytosolic N terminus that contains two tetratricopeptide-repeats (TPRs). Fis1 was initially identified in *Saccharomyces cerevisiae* as a protein required for the recruitment of the yeast Drp1 homolog, Dnm1, during mitochondrial fission.[1] A more complex functional redundancy and specialization of Drp1 mitochondrial receptors has evolved in animal cells.[2–4] Thus, in mammalian cells, the absence of Fis1 does not impair mitochondrial fission to the same extent as the absence of Drp1.[1,3,5–9] The marginal role Fis1 plays in animal mitochondrial fission, however, contrasts with the lethality its systemic genetic ablation causes in mice.[10,11] In human cells, Fis1 interacts with the Tre-2/Bub2/Cdc16 family member 15 (TBC1D15),[12] a Rab7A GTPase-activating protein (Rab-GAP).[13,14] Recruitment of TBC1D15 by Fis1 to the mitochondria modulates Rab7A activity during both the PINK-Parkin-mediated mitophagy[7] and the remodeling of mitochondria-lysosome contacts.[15,16] Consistent with findings in human cells,[6,7] the specific deletion of Fis1 in mouse skeletal muscle causes aberrant mitophagosome formation and is also associated with an increased synthesis of cytokines in animals subjected to endurance exhaustive exercise.[10] Overexpression of Fis1 in mice subjected to a high-fat diet decreased the synthesis of type I interferons[17] suggesting Fis1 plays a role in regulating inflammatory responses through an unknown mechanism. Further, Fis1 and TBC1D15 were identified among uncharacterized hits the absence of which increased the activation of a reporter driven by interferon-stimulated response elements (ISRE) in a genome-wide CRISPR-interference screening for changes in the activation of the innate immune cyclic dinucleotide sensor stimulator of interferon genes (STING).[18]

STING is a transmembrane endoplasmic reticulum innate immune receptor[19] that traffics to the Golgi upon binding to cyclic-dinucleotides[20–23] of either bacterial origin or endogenously synthesized in response to double-stranded DNA (dsDNA) by the 2′3′-guanosine monophosphate-adenosine monophosphate dinucleotide (cGAMP) synthase (cGAS).[24,25] Then, STING acts as a scaffold, mediating the interaction between the interferon regulatory factor 3 (IRF3) and the TANK-binding kinase 1 (TBK1). IRF3 phosphorylation by TBK1 is required to trigger the synthesis of type I interferons.[19,26,27] Active STING is trafficked from the endoplasmic reticulum (ER) through the Golgi to Rab7A-containing endolysosomal compartments for its degradation.[28–31] Basal STING protein levels are also regulated through lysosomal degradation during homeostatic conditions downregulating the tonic activation of the pathway.[32]

We characterized the mechanism underlying the relationship between STING-mediated signaling and mitochondrial Fis1 in

This is an open access article under the CC BY license (http://creativecommons.org/licenses/by/4.0/).

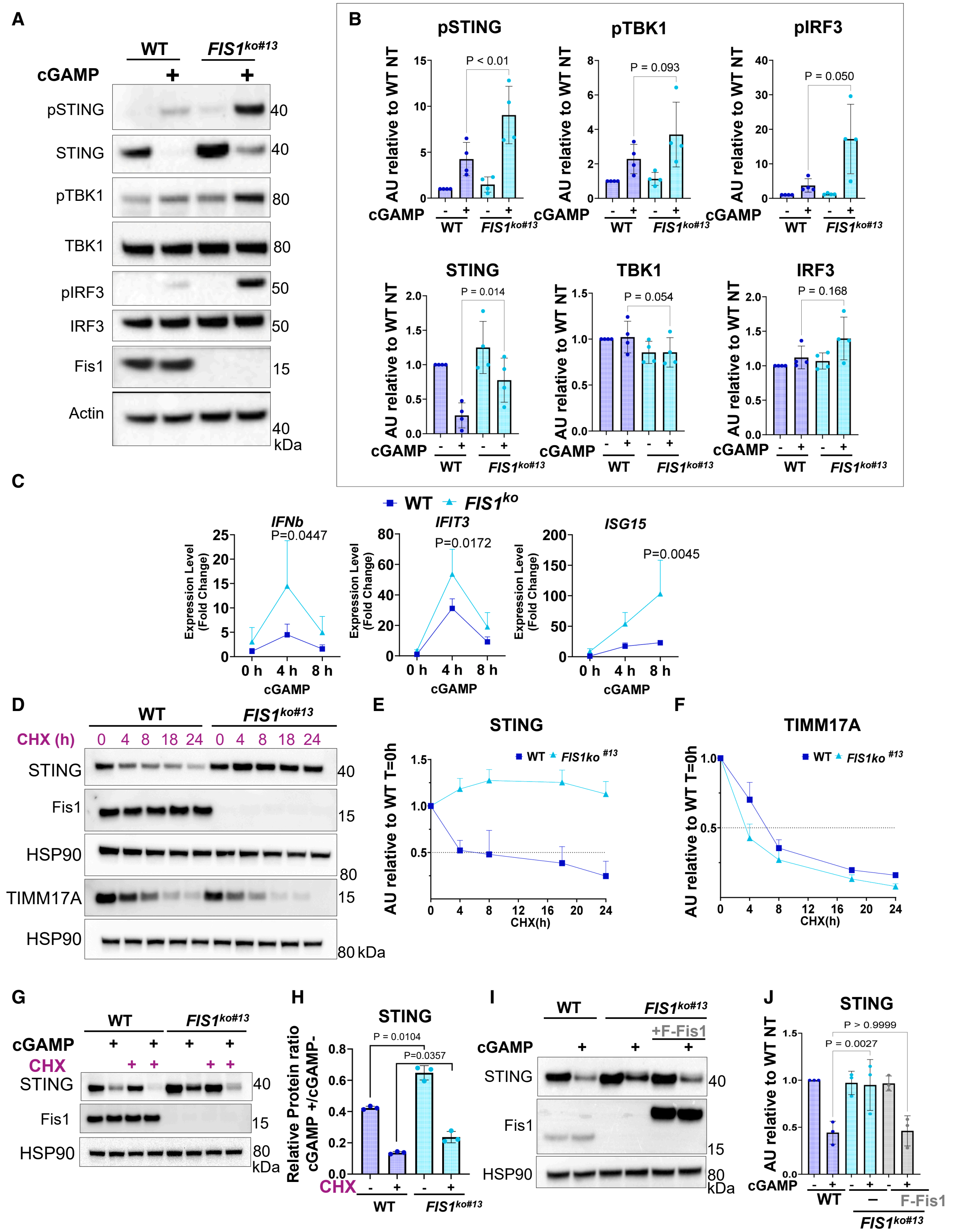

(legend on next page)

HeLa cells and found that the absence of Fis1 increases basal and active STING stability, boosting the downstream signaling induced by cGAMP. STING ER to Golgi trafficking follows similar dynamics in wild type (WT) and *FIS1*^{ko} cells causing Golgi cisternae swelling. However, active STING delivery to Rab7A-positive vesicles decreases in cells lacking Fis1. Consistently, STING degradation is impaired in cells lacking the Rab7A-GAP TBC1D15 or Rab7A. In the absence of Fis1, TBC1D15 is not recruited to the mitochondria and its interaction with Rab7A is hampered, unbalancing Rab7A GTPase activity. We propose that mitochondrial tethered Fis1 is required to recruit and tune the affinity of TBC1D15 for Rab7A, thus ensuring proper trafficking of activated STING to late endosomes. Our data unveil a mechanism by which mitochondria can influence Rab7A activity beyond local processes through the Fis1-TBC1D15 axis.

RESULTS

The absence of Fis1 increases STING stability in HeLa cells

To test whether the absence of Fis1 could alter STING-TBK1-IRF3 signaling, we compared the phosphorylation status of these proteins in WT and Fis1 knock-out (*FIS1*^{ko}) HeLa cells after treatment with cGAMP or with no treatment (NT). The phosphorylation levels of STING and IRF3 were higher upon cGAMP treatment in cells lacking Fis1 compared to WT-treated cells (Figures 1A and 1B). Consistently, the abundance of *IFNb*, *IFIT3*, and *ISG15* transcripts was enhanced (Figure 1C) in the absence of Fis1. After cGAMP treatment, STING protein levels decreased in both WT and *FIS1*^{ko} due to degradation at endolysosomal compartments. However, compared to WT cells, STING protein levels remained higher in *FIS1*^{ko} cells treated with cGAMP (Figures 1A and 1C). We assayed STING protein stability using a cycloheximide (CHX) pulse-chase assay under basal growth conditions and found that STING half-life($T_{1/2}$) in WT cells was around 12.6 h, whereas no decrease on STING protein levels was detected in *FIS1*^{ko} cells after 24 h of CHX treatment (Figures 1D and 1E; Figure S1M). As a control, we followed TIMM17A protein stability and observed that its half-life ($T_{1/2}$) was similar in WT and *FIS1*^{ko} cells (WT $T_{1/2\sim}$9.02 h, *FIS1*^{ko} $T_{1/2\sim}$7.1 h) (Figures 1D–1F; Figure S1N). When cells were treated with cGAMP at the same time that protein synthesis was inhibited with CHX, we observed increased STING stability in cells lacking Fis1 (Figures 1G and 1H).

We confirmed the increased STING stability caused by the absence of Fis1 in a different *FIS1*^{ko} clone (Figures S1A and S1B) and in *FIS1*^{ko} HCT116 cells (Figures S1E and S1F). Further, we observed that the increased STING stability phenotype caused by the absence of Fis1 was rescued by reintroducing 3xFLAG-Fis1 (F-Fis1) but not by introducing the empty vector (Figures 1I and 1J; Figures S1C and S1D).

To rule out a crosstalk between STING activation and mitophagy, we tested if activation of STING via cGAS-synthesized cGAMP or by directly adding cGAMP to WT HeLa$^{+mCherry\text{-}Parkin}$ cells could induce PINK1 stabilization. We detected PINK1 stabilization only upon mitochondria depolarization with antimycin and oligomycin (AO) and not when cells were transfected with dsDNA or treated with cGAMP, suggesting STING activation does not induce mitophagy (Figure S1G).

To assess whether Fis1 membrane anchoring was required for the proper degradation of activated STING, we generated a cytosolic version of Fis1 truncating amino acids (aa) 121–152 (Fis1$^{\Delta C}$). We found that the expression of Fis1$^{\Delta C}$ in *FIS1*^{ko} cells did not rescue the phenotype caused by the absence of Fis1 (Figures S1H–S1K). We also tested a chimeric form of Fis1 exclusively targeted to the mitochondria, in which we replaced the Fis1 C-tail with the transmembrane helix (aa 101–135) of the mitochondrial protein OMP25 (Fis1$^{\Delta C\text{-}OMP25}$) and observed that cells expressing this Fis1 form displayed a similar ratio of STING degradation as when WT Fis1 was reintroduced (Figures S1J and S1K). The subcellular localization of all the herein described Fis1 constructs was corroborated by confocal microscopy (Figure S1L).

Trafficking of STING to Rab7A-positive vesicles is hindered in cells lacking Fis1

We treated with cGAMP WT and *FIS1*^{ko} cells stably expressing mEGFP-STING and followed STING trafficking from the ER to the Golgi. Immunofluorescent detection of the protein disulfide isomerase (PDI) (Figure S2) and the *trans*-Golgi network 46 protein (TGN46) (Figure 2A) was used to label the ER and the Golgi, respectively. In both WT and *FIS1*^{ko} cells, STING trafficking dynamics upon a cGAMP treatment were similar (Figures 2A–2C; Figure S2). We detected only a slightly higher

Figure 1. Fis1 absence stabilizes STING

(A) Immunoblot for phosphorylated and total STING, TBK1, and IRF3 proteins from WT or *FIS1*$^{ko\#13}$ whole-cell lysates. Cells were stimulated with cGAMP or left untreated. Actin was blotted as a loading control.

(B) Graphs and statistical analysis of the phosphorylated and total protein levels in (A) (AU, arbitrary units; NT, no treatment; $n = 4$, mean ± SD, two tailed, paired t test: WT + cGAMP vs. *FIS1*$^{ko\#13}$+cGAMP, P = p value).

(C) Relative mRNA expression levels of *IFNb*, *IFIT3*, and *ISG15* in WT and *FIS1*$^{ko\#13}$ cells detected by qPCR after the indicated times of treatment with cGAMP ($n = 3$, $2^{-\Delta\Delta Ct}$ values mean ± SD, 2-way ANOVA with a Sidak's post-hoc test, P = p value).

(D) Immunoblot of STING and TIMM17A from lysates of WT or *FIS1*$^{ko\#13}$ cells treated for the indicated times with cycloheximide (CHX). HSP90 was detected as a loading control.

(E and F) Plots of normalized (E) STING and (F) TIMM17A protein levels quantified from (D) (AU, arbitrary units; NT, no treatment, $n = 3$, mean ± SD).

(G) STING protein levels detected by immunoblot after 8 h of treatment with cGAMP or cGAMP +CHX. HSP90 was blotted as loading control.

(H) Graph of STING protein levels in (G). The ratio of STING with cGAMP to that without cGAMP (cGAMP+/cGAMP-) was plotted ($n = 3$, mean ± SD, paired t test: WT + vs. *FIS1*$^{ko\#13}$, P = p value).

(I) Immunoblot of STING in WT, *FIS1*$^{ko\#13}$, and *FIS1*$^{ko\#13}$+3xFLAG-Fis1 HeLa cells treated with cGAMP or left with no treatment (NT). Fis1 expression was assayed and Hsp90 was included as a loading control.

(J) STING protein levels from (I), graphed and statistically analyzed ($n = 3$, mean ± SD, one-way ANOVA post-hoc Bonferroni, P = p value).

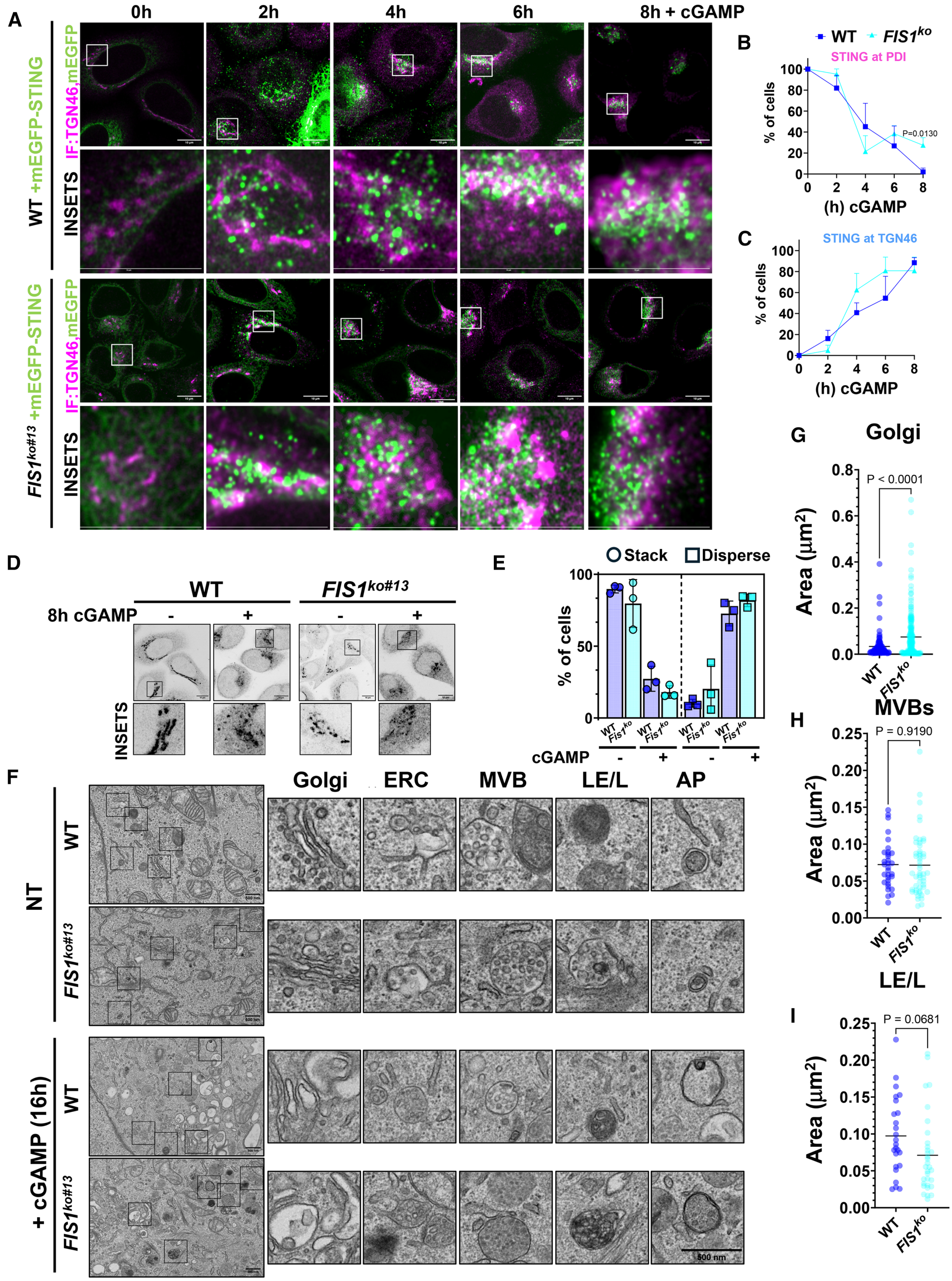

Figure 2. STING ER-to-Golgi trafficking is unaffected in *FIS1*^{ko} HeLa cells

(A) Confocal microscopy analysis of mEGFP-STING subcellular localization in WT and *FIS1*$^{ko\#13}$ HeLa cells. Cells were treated with cGAMP for the indicated times (h) and then were prepared for immunofluorescent detection of TGN46. Scale bars: 10 μm. STING and TGN images were acquired independently and were merged and adjusted. To ease visualization, inset images were smoothed and contrast was adjusted to a different extent in each part of the image.

(legend continued on next page)

percentage of cells with STING signal at the ER after 8 h of treatment with cGAMP in *FIS1*^{ko} cells (Figure 2B). We noticed that the TGN46 signal changed upon activation of STING with cGAMP, shifting from being restricted in clear Golgi stacks to becoming dispersed and diffused throughout the perinuclear region (Figures 2D and 2E). We assessed the cell ultrastructure changes caused by activating STING with cGAMP in WT and *FIS1*^{ko} cells using transmission electron microscopy (TEM). We detected that the Golgi morphology changed drastically in both WT and *FIS1*^{ko} cells as a consequence of STING activation (Figure 2F). Upon cGAMP treatment, Golgi stacks swell and were dispersed through the perinuclear region, similar to the observations of TGN46 immunodetection (Figure 2D). The Golgi stacks, including dilated cisternae, had a larger area in cells lacking Fis1 compared to WT (Figure 2G), while the area of multivesicular bodies (MVBs) (Figure 2H) and vesicles containing electron dense material such as late endosomes and lysosomes (LE/L) was similar in WT and *FIS1*^{ko} cells (Figure 2I).

We followed the trafficking of mEGFP-STING to mCherry-Rab7A-positive vesicles (Figure 3A; Figure S3) and observed an increase in the percentage of mEGFP-STING within mCh-Rab7A signal that peaked in WT cells after 6 and 8 h of cGAMP treatment. The percentage of mEGFP-STING signal detected with mCh-Rab7A vesicles remained stable in *FIS1*^{ko} cells and did not increase upon a cGAMP treatment (Figures 3A and 3B). Congruently with previous reports,[28,33] we observed that knocking down (kd) RAB7A impaired STING degradation significantly (Figures 3C and 3D). A slight increase in the percentage of mEGFP-STING colocalization (from 1.49% to 2.43%) with mCh-Rab5A positive early endosomes was observed in WT cells after 8 h of STING activation with cGAMP, this was not detected in *FIS1*^{ko} cells (Figures 3E and 3F). The mEGFP-STING signal within mCh-Rab11-positive regions increased similarly in both WT and *FIS1*^{ko} cells (Figures 3G and 3H). We knocked down *RAB5A* and *RAB11*, decreasing their expression by ~32.1% and by ~45.09%, respectively, and did not observe any changes in STING degradation (Figures S3B, S3C, S3D, and S3E).

We detected the delivery of mEGFP-STING to lysosomes using immunofluorescent detection of LAMP1. We assessed the delivery of mEGFP-STING to lysosomes through an assay commonly used to measure the delivery of cargoes to lysosomes when assaying autophagy flux[34]; treating the cells for 8 h with cGAMP or with cGAMP and chloroquine (CQ), which increases lysosomal pH and decreases lysosomal degradation. The mEGFP-STING signal in LAMP1 vesicles was low in both WT and *FIS1*^{ko} cells after 0 and 8 h of treatment with cGAMP (Figures 3I and 3J). However, the mEGFP-STING signal accumulated in LAMP1-positive vesicles in cells treated with cGAMP plus CQ in WT cells, while the absence of Fis1 halted this accumulation (Figures 3I and 3J).

STING degradation is impaired in cells lacking the Rab7A-GAP and Fis1 interactor TBC1D15

Given that we observed altered trafficking of STING to Rab7A-positive vesicles in the absence of Fis1, we tested whether avoiding Fis1-TBC1D15 interaction, by deleting the region of Fis1 required to interact with TBC1D15, impaired STING degradation. STING was degraded to a similar extent upon cGAMP treatment in WT cells and in *FIS1*^{ko} expressing an mEGFP-tagged WT form of Fis1. When a mutant form of Fis1 lacking aa 77–98 (the region of Fis1 required to recruit TBC1D15)[12,35] was stably expressed in *FIS1*^{ko} cells, a decreased rate of STING degradation was observed, comparable to the rate observed in *FIS1*^{ko} cells (Figures 4A and 4B). The Δ77-98 truncation impaired the detection of the protein by Fis1 antibody (Figure 4A), but we corroborated its expression and mitochondria localization by detecting the mEGFP tag (Figures 4A and 4C).

We examined STING protein levels in HeLa *TBC1D15*^{ko} cells and found, similar to *FIS1*^{ko} cells, that cGAMP-activated STING stability was higher compared to WT cells (Figures 4D and 4E). This increased stability of active STING in *TBC1D15*^{ko} was associated with increased STING phosphorylation (Figures 4D and 4E).

TBC1D15 and TBC1D17 are functionally redundant during mitophagy[7]; however, we observed that the absence of TBC1D17 in HeLa cells did not increase active STING stability (Figures 4F and 4G), suggesting that despite TBC1D17 and TBC1D15 protein sequences share around 50% identity, they have evolved non-overlapping functions.

The observed stabilization of active STING in cells lacking TBC1D15 was validated in a different KO clone (Figures S2A and S2B). In addition, we reintroduced an mEGFP-tagged form of TBC1D15 isoform 1 in to *TBC1D15*^{ko} cells and observed that the stabilization of cGAMP-activated STING was rescued (Figures 4H and 4I), while we did not observe rescue of the phenotype by introducing the empty vector (Figures S2C and S2D).

We expressed mEGFP-TBC1D15 into cells lacking both *FIS1* and *TBC1D15* (*FIS1/TBC1D15*^{dko}), its expression did not rescue the increased stabilization of active STING observed in the double knock out compared to WT cells, suggesting *FIS1* displays upstream epistasis to *TBC1D15* (Figures 4J and 4K).

To test whether the hyperactivation of STING in cells lacking Fis1 or TBC1D15 could be due to impaired mitochondrial homeostasis, we imaged immunofluorescently stained mitochondria in WT, *DRP1*^{ko}, *FIS1*^{ko}, and *TBC1D15*^{ko} cells (Figures S2E–S2J).

(B and C) Quantification of the percentage of cells showing STING colocalization with the ER marker PDI or (C) with the trans-Golgi network marker TGN46 from images in A (n = 3, mean ± SD, multiple t tests: WT vs. *FIS1*$^{ko\#13}$, P = p value).

(D) Immunofluorescence staining of TGN46 in WT and *FIS1*$^{ko\#13}$ treated with cGAMP for 8 h or left untreated. Scale bars: 10 μm.

(E) Quantification of Golgi morphology parameters from images as in (D) (n = 3, mean ± SD).

(F) Transmission electron microscopy (TEM) imaging of WT and *FIS1*$^{ko\#13}$ HeLa cells (10 cells per condition) treated with cGAMP or left untreated. Golgi, ERC (endosome recycling compartment), MVB (multivesicular bodies), LE/L (late endosomes/lysosomes), and AP (autophagosomes). Scale bars: 500 nm.

(G–I) Quantifications of the area of (G) Golgi stacks, (H) MVB, or (I) LE/L from WT or *FIS1*$^{ko\#13}$ HeLa cells treated with cGAMP. (n = 10, mean ± SD, two-tailed unpaired t test: WT vs. *FIS1*$^{ko\#13}$, P = p value).

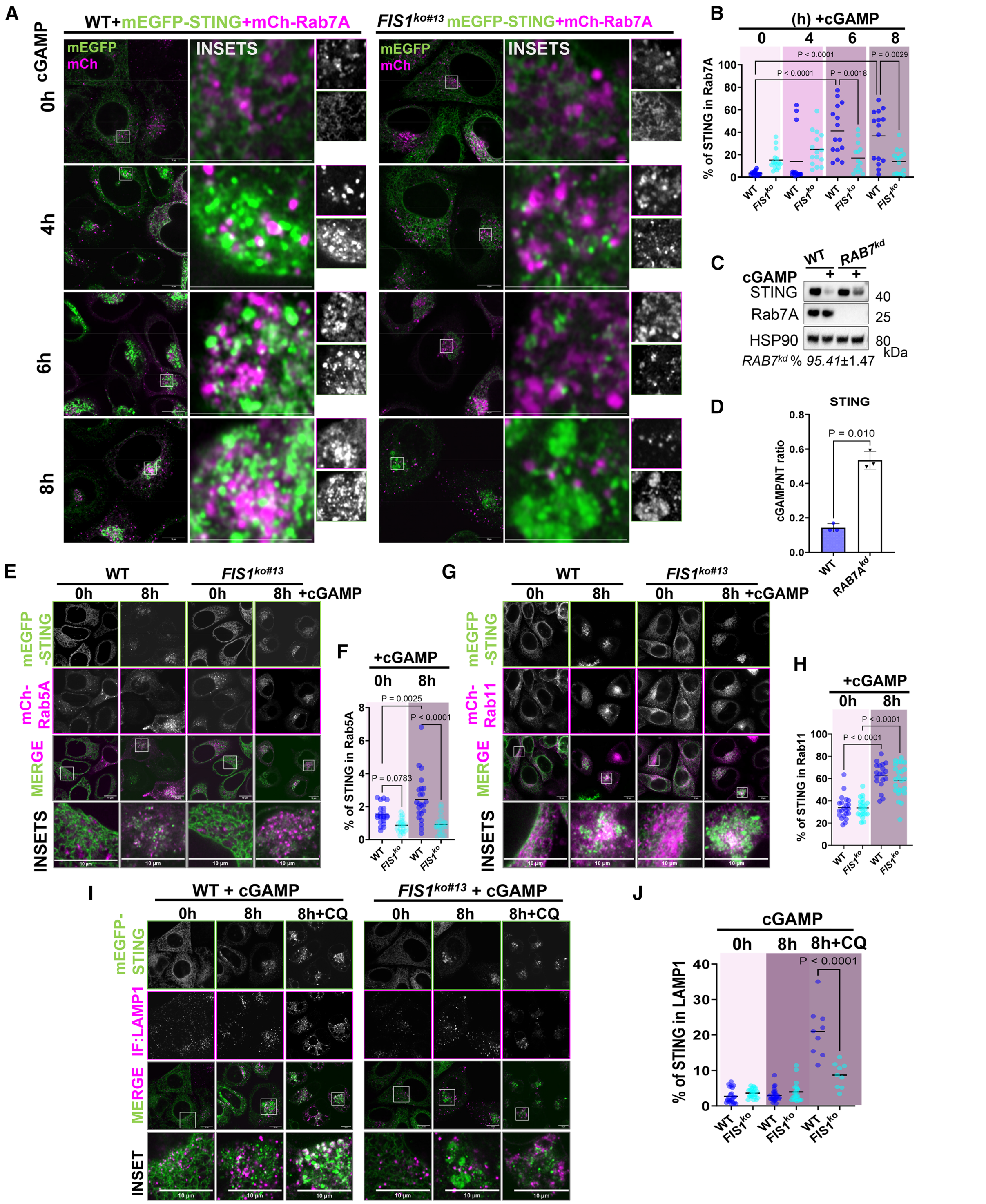

Figure 3. Lack of Fis1 hinders STING trafficking to late endosomes and lysosomes

(A) Confocal microscopy time-course analysis of mEGFP-STING and mCherry-Rab7 in WT or *FIS1*$^{ko\#13}$ cells treated with cGAMP.

(B) Quantification of STING within Rab7-positive areas from (A) (*n* = 15 cells/condition, mean ± SD, two-way ANOVA, post-hoc Bonferroni, P = *p* value).

(C) Immunoblot analysis of STING in WT and *RAB7A*^{kd} cells upon a cGAMP treatment. Rab7A expression was assessed and HSP90 was used as loading control.

(D) Densitometric quantification of immunoblot signals from (C) (*n* = 3, mean ± SD, paired two-tailed *t* test: WT vs. *RAB7A*^{kd}, P = *p* value).

(E) Representative images of mEGFP-STING and mCherry-Rab5.

(F) Quantification of STING within Rab5-positive vesicles from (E) (*n* = 22, 24, 23, 24 cells, mean ± SD, two-way ANOVA, Bonferroni, P = *p* value).

(legend continued on next page)

As previously reported,[2–5,7] the absence of Drp1 caused a phenotype with elongated and swollen joined mitochondria (Figure S2F). In contrast, the absence of Fis1 or TBC1D15 did not cause any similar apparent alterations in mitochondrial morphology (Figures S2G–S2J). We tested if the hyperactivation of STING in the absence of Fis1 or TBC1D15 could be due to impaired mitophagy leading to increased cGAS activation. Intracellular cGAMP was measured by ELISA after inducing mitophagy with AO in WT, *FIS1*^{ko}, or *TBC1D15ko* cells, all expressing Parkin (mCh-PRKN). Mitophagy induction did not increase cGAS activity upon AO treatment in any of the tested conditions (Figure S2K).

TBC1D15 subcellular localization is influenced by Fis1 expression and by STING activation

To evaluate whether TBC1D15 subcellular localization was altered by the expression of mutant forms of Fis1, we tracked by confocal microscopy an mEGFP-tagged form of TBC1D15 expressed in *FIS1/TBC1D15*^{dko} cells that also expressed either WT Fis1 or mutant forms of the protein. In agreement with previous reports,[7,12,36] TBC1D15 localization was influenced by Fis1 expression levels and localization (Figures 5A–5C; Figure S5). When WT Fis1 was stably expressed, TBC1D15 was enriched at the mitochondria (Figures 3A and 3B; Figures S5A, S5B, S5E, and S5F). While when cytosolic Fis1$^{\Delta C}$ was expressed, TBC1D15 was detected mainly in the cytoplasm with Fis1 (Figures S5A and S5B).

In the absence of Fis1 expression, TBC1D15 localized either at the cytosol or accumulated at the perinuclear region of cells, in close vicinity to the markers Golgi matrix protein130 (GM130) (Figures 5C and 5D) and TGN46 (Figures 5E and 5F; Figures S5D, S5E, and S5G). When a mutant form of Fis1 lacking the region that interacts with TBC1D15 (Fis1$^{\Delta 77\text{-}98}$) was expressed, mCh-TBC1D15 displayed very low colocalization with the mitochondria marker TOMM20 and accumulated at the perinuclear region of cells in close vicinity to the Golgi marker TGN46 (Figures S5E–S5G).

We then followed the subcellular localization of mScarlet-tagged (mSca) STING stably expressed in *FIS1/TBC1D15*^{dko} cells in which Fis1^{WT} and mEGFP-TBC1D15 were reintroduced (*FIS1/TBC1D15*^{dko}+mEGFP-TBC1D15+mSca-STING+Fis1). We acquired images of fixed cells after either 4 or 6 h of cGAMP treatment or left with NT. Consistent with the described passage of STING through the Golgi during its activation[20–23] and our previous results (Figures 2A and 2B), we observed that mSca-STING redistributed from the ER to the perinuclear region of cells after being exposed to cGAMP (Figure 5A). We also observed that upon STING activation with cGAMP, the subcellular localization of mEGFP-TBC1D15 shifted from the mitochondria to the perinuclear region of these cells (*FIS1/TBC1D15*^{dko} +mEGFP-TBC1D15+mSca-STING+Fis1). The percentage of cells in which TBC1D15 accumulated at mitochondria decreased over time following cGAMP treatment (Figures 5A and 5B). After 8 h of treatment with cGAMP, we observed in WT cells an increase of mEGFP-TBC1D15 signal localized within the signal of both GM130 and TGN46 Golgi markers(Figures 5C–5F). In the absence of Fis1 expression, the proportion of TBC1D15 signal detected within the GM130 marker decreased upon 8 h of treatment with cGAMP but remained localized surrounding it (Figures 5C and 5D). A similar proportion of TBC1D15 accumulated in regions positive to TGN46 in *FIS*^{ko} cells, irrespective of the treatment (Figures 5E and 5F).

We detected a slight increase in the percentage of TBC1D15 signal within the lysosomal marker LAMP1 signal in the presence of Fis1, which was not observed in its absence (Figures 5G and 5H).

Fis1 absence hampers Rab7A interaction with TBC1D15, impairing Rab7A GTPase activity

Given that TBC1D15 is the main Rab7A-GAP and that the absence of, either or both, Fis1 and TBC1D15 caused a similar increase on STING stability upon cGAMP activation, we hypothesized that Fis1 absence could be involved in regulating the TBC1D15-mediated stimulation of Rab7A GTPase activity. To test this hypothesis, we assessed if Fis1 was required for the association between TBC1D15 and Rab7A. We immunoprecipitated (IP) an HA-tagged form of Rab7A that was stably expressed in WT and *FIS1*^{ko} cells (Figure 6A) and tested for TBC1D15 co-immunoprecipitation. When we analyzed the eluted HA-bound fraction by immunoblotting against TBC1D15, we found that the absence of Fis1 caused a decrease in the co-immunoprecipitation of TBC1D15 with HA-Rab7A in lysates from both cGAMP-treated and -untreated cells (Figures 6A and 6B). We then evaluated if the observed decreased association between Rab7A and TBC1D15 imbalanced Rab7A GTPase activity by immunoprecipitating GTP-bound Rab7A with a specific antibody against this form of the protein. In accordance with the positive control of WT cell lysates incubated with GTP, which stimulated GTP loading into Rab7A (Figure 6C), an increased level of Rab7A protein was immunodetected when GTP-Rab7A was pulled down from lysates of *FIS1*^{ko} cells in all the conditions tested. We corroborated that the absence of Fis1 in these conditions increased STING stability assaying whole cells lysates (Figure 6D).

Rab7A S72 phosphorylation (pRab7A) has been suggested to contribute to Rab7A GTPase activity regulation,[37] based on the observation that the overexpression of a phosphomimetic S72E form of Rab7A delays EGFR degradation.[38] Further, TBK1 phosphorylates Rab7A S72 during STING activation,[39] while the overexpression of S72E Rab7A impairs active STING degradation,[40] suggesting Rab7A phosphorylation may be regulated during STING trafficking. We analyzed by immunoblot the levels of Rab7A S72 phosphorylation and observed an increase of S72 pRab7A induced by activation of STING with

(G) Confocal microscopy of mEGFP-STING and mCherry-Rab11-positive compartments.
(H) Quantification of STING within Rab11-positive signal from (G) (n = 22, 21, 19, 26 cells, mean ± SD, two-way ANOVA, post-hoc Bonferroni, P = p value).
(I) Confocal microscopy images of mEGFP-STING and LAMP1-positive lysosomes in the absence or presence of chloroquine (CQ).
(J) Quantification of STING accumulation within LAMP1 signal from (I) (n = 24, 24, 27, 20, 9 cells, mean ± SD, one-way ANOVA, Šídák's, P = p value). To ease visualization of confocal imaging, the contrast was adjusted to a different extent in each part of the image. Scale bars for all imaging: 10 μm.

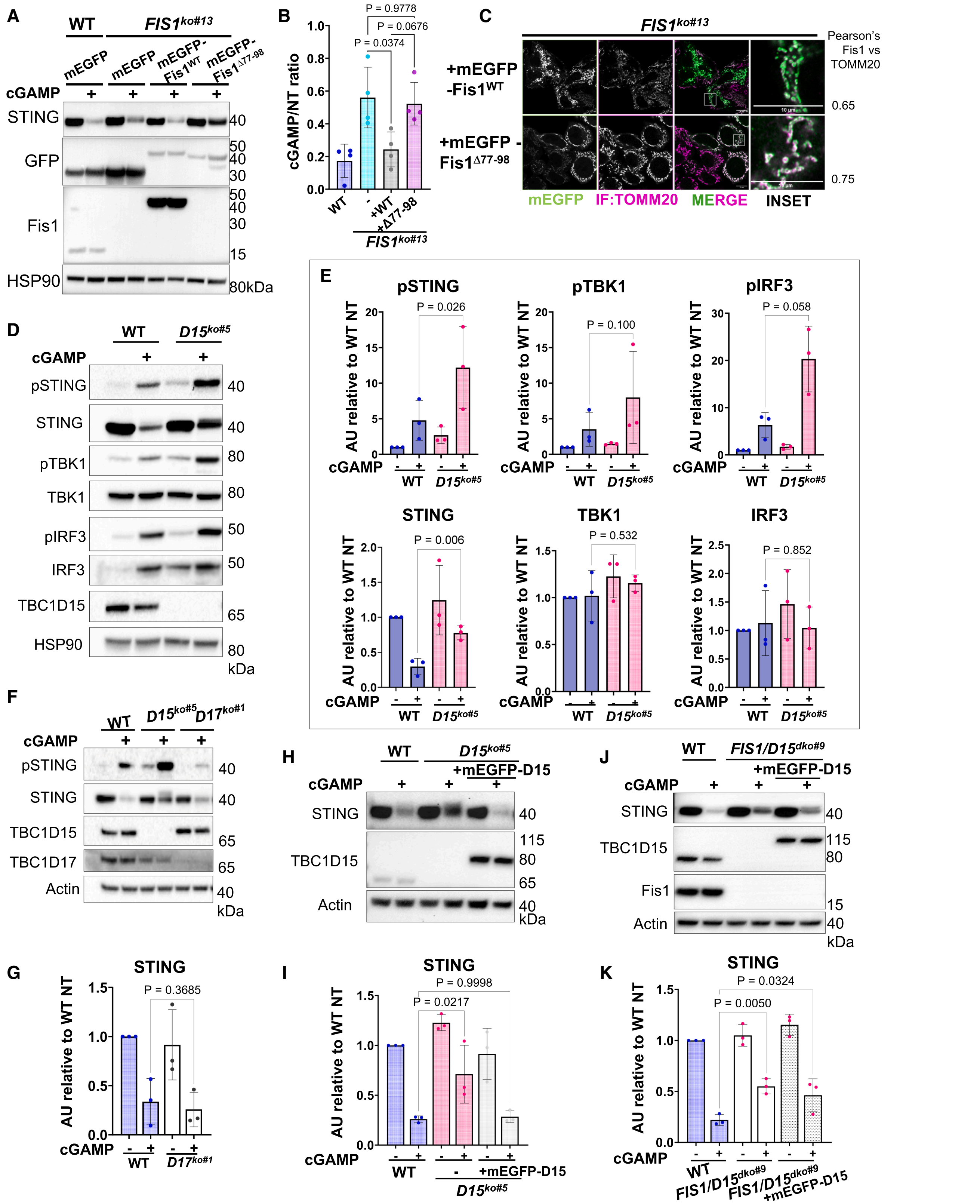

(legend on next page)

CellPress
OPEN ACCESS

cGAMP in WT cells (Figures 6E–6G). The absence of Fis1 caused higher basal levels of pRab7A (Figures 6E and 6F), congruent with the observed increase of immunoprecipitated GTP-bound Rab7A. The stable overexpression of a GTP-locked form of Rab7A (HA-Rab7A^{Q67L})[41,42] caused a slight increase in active STING stability in *FIS1^{ko}* cells (approximately a 0.26 increase) (Figures 6G–6I). Together, our data suggest that the reduced affinity between TBC1D15 and Rab7A, caused by the absence of Fis1, dampens Rab7A GTPase activity.

DISCUSSION

Mitochondria play a key role in innate immune signaling through the mitochondria antiviral signaling protein (MAVS) and through activation of the cGAS-STING pathway by mitochondrial DNA (mtDNA).[43] In this work, we report a mechanism by which mitochondria can influence STING signaling independent of mtDNA. We found that the absence of the mitochondrial protein Fis1 in HeLa cells stabilizes STING, boosting its downstream signaling when activated by cGAMP possibly due to an increased initial pool of STING and due to a decreased degradation rate of active STING. The increased levels of cytokines transcription that we observed in HeLa cells when Fis1 was absent suggest that decreased Fis1 expression could impair systemic STING downstream signaling and could be the underlying cause of the previously reported links between Fis1 and inflammation in mice.[10,17]

Our data show that STING trafficking through the Golgi changes its structure; specifically, we observed it causes dispersion and dilation of the cisternae. Dilation of the Golgi cisternae could be observed when the secretory capacity of cells is exceeded or compromised.[44,45] Also, changes in the Golgi structure have been reported when the proton gradient, increasing from *cis* to *trans*, is lost upon treatment with the Na+/H+ ionophore monensin,[46–48] due to expression of viral ion channels[49,50] or in the absence of the Golgi pH regulator anion channel (GPHR).[51] Large STING oligomers are assembled during STING activation at the ER[52] and are trafficked to the Golgi as large cargoes possibly dilating the Golgi cisternae during its passage. Further, it has been proposed that STING could act as a proton channel,[53,54] which could imbalance the ion equilibrium of Golgi cisternae similarly to other agents that disturb the Golgi pH gradient, contributing to the swelling of the Golgi stacks.

The area of Golgi stacks in cells lacking Fis1 was larger compared to WT cells, which could be due to an imbalanced trafficking flow of STING. Recently, the absence of TAX1BP1 has been reported to impair STING endolysosomal degradation and also Golgi morphology.[55]

Similar to the phenotype of mutations in proteins required for STING traffic from the TGN to endolysosomes,[29–31,56–59] the absence of Fis1 does not impair STING trafficking from the ER to the Golgi, suggesting its absence hampers a later stage of STING trafficking. Congruently, a lesser percentage of STING accumulated in Rab7A-positive vesicles in the absence of Fis1 after more than 6 h of activation with cGAMP. Furthermore, lack of Fis1 hampers STING delivery to lysosomes, suggesting it is required for proper lysosome-mediated STING degradation.

Fis1 interacts with the Rab7A-GAP TBC1D15 recruiting it to mitochondria.[12] The Fis1-TBC1D15 interaction is required locally during mitophagy[7] and has been suggested to control the untethering of mitochondria-lysosome contacts.[15] On the other hand, our data show that TBC1D15 localization shifts from Fis1-mediated attachment to the mitochondria to become enriched at the Golgi upon STING activation with cGAMP. This is consistent with the identification of TBC1D15 among enriched proteins in proximity to STING at late stages of its trafficking.[29] In the absence of Fis1, TBC1D15 is constitutively located at the Golgi and decreases its colocalization with the *cis*-Golgi marker GM130 upon cGAMP treatment, either due to impaired regulation of its localization during STING trafficking or due to the increased dilation of the Golgi stacks.

As observed when Rab7A expression is dampened, the absence of TBC1D15 halts STING degradation, suggesting

Figure 4. STING degradation is impaired in cells lacking Fis1 interactor TBC1D15

(A) STING degradation assayed by immunoblot in WT, *FIS1$^{ko\#13}$*, and *FIS1$^{ko\#13}$* cells stably expressing WT Fis1 or the Δ77-98 Fis1 mutant following stimulation with cGAMP. Expression of constructs was verified by blotting vs. GFP and Fis1. HSP90 was used as loading control.

(B) STING degradation ratio quantified from immunoblot signals as in (A) (NT, no treatment, $n = 4$, mean ± SD, one-way ANOVA, Šídák's, P = p value).

(C) Confocal microscopy assessing mitochondrial localization (TOMM20) of WT FIS1 and the Δ77-98 mutant. Colocalization assayed by Pearson's correlation Fis1 vs. TOMM20. Scale bars: 10 μm. To ease visualization, images were smoothed and contrast was adjusted to a different extent in each part of the image.

(D) Immunoblot of STING, phosphorylated STING, and downstream signaling components in WT and *TBC1D15$^{ko\#5}$* cells with no treatment or stimulated with cGAMP. HSP90 was included as a loading control.

(E) Quantification of phosphorylated and total STING and downstream components of experiments as in (D) (NT, no treatment, AU, arbitrary units, $n = 3$, mean ± SD, two-tailed paired t test: WT + cGAMP vs. *TBC1D15$^{ko\#5}$*+cGAMP, P = p value).

(F) WT, *TBC1D15$^{ko\#5}$*, or *TBC1D17$^{ko\#1}$* cell lysates immunoblotted for phosphorylated and total STING from untreated or cGAMP-stimulated cells. Blotting against TBC1D15 and TBC1D17 was included. Actin was detected as a loading control.

(G) Quantification of total STING protein levels in WT vs. *TBC1D17$^{ko\#1}$* cells as in (F) (NT, no treatment; AU, arbitrary units, $n = 3$, mean ± SD, two-tailed paired t test, WT + cGAMP vs. *TBC1D17$^{ko\#1}$*+cGAMP, P = p value).

(H) STING and TBC1D15 detected in WT, *TBC1D15$^{ko\#5}$*, or *TBC1D15$^{ko\#5}$* cells stably expressing mEGFP-TBC1D15. Cells were stimulated with cGAMP or left with no treatment (NT). Actin was blotted as a loading control.

(I) Plots of densitometric quantifications of experiments as in (H) (NT, no treatment; AU, arbitrary units, $n = 3$, mean ± SD, one-way ANOVA, Bonferroni, P = p value).

(J) STING protein levels immunoblotted in WT, *FIS1/TBC1D15$^{dko\#9}$*, and *FIS1/TBC1D15$^{dko\#9}$*+mEGFP-TBC1D15 cells stimulated with cGAMP or with no treatment (NT). Fis1 and TBC1D15 were blotted, and Actin was included as a loading control.

(K) Quantification and statistical analysis of total STING protein levels as in (J) (NT, no treatment; AU, arbitrary units, $n = 3$, mean ± SD, one-way ANOVA, Bonferroni). *TBC1D15$^{ko\#5}$ = D15$^{ko\#5}$*; *TBC1D17$^{ko\#1}$ = D17$^{ko\#1}$*.

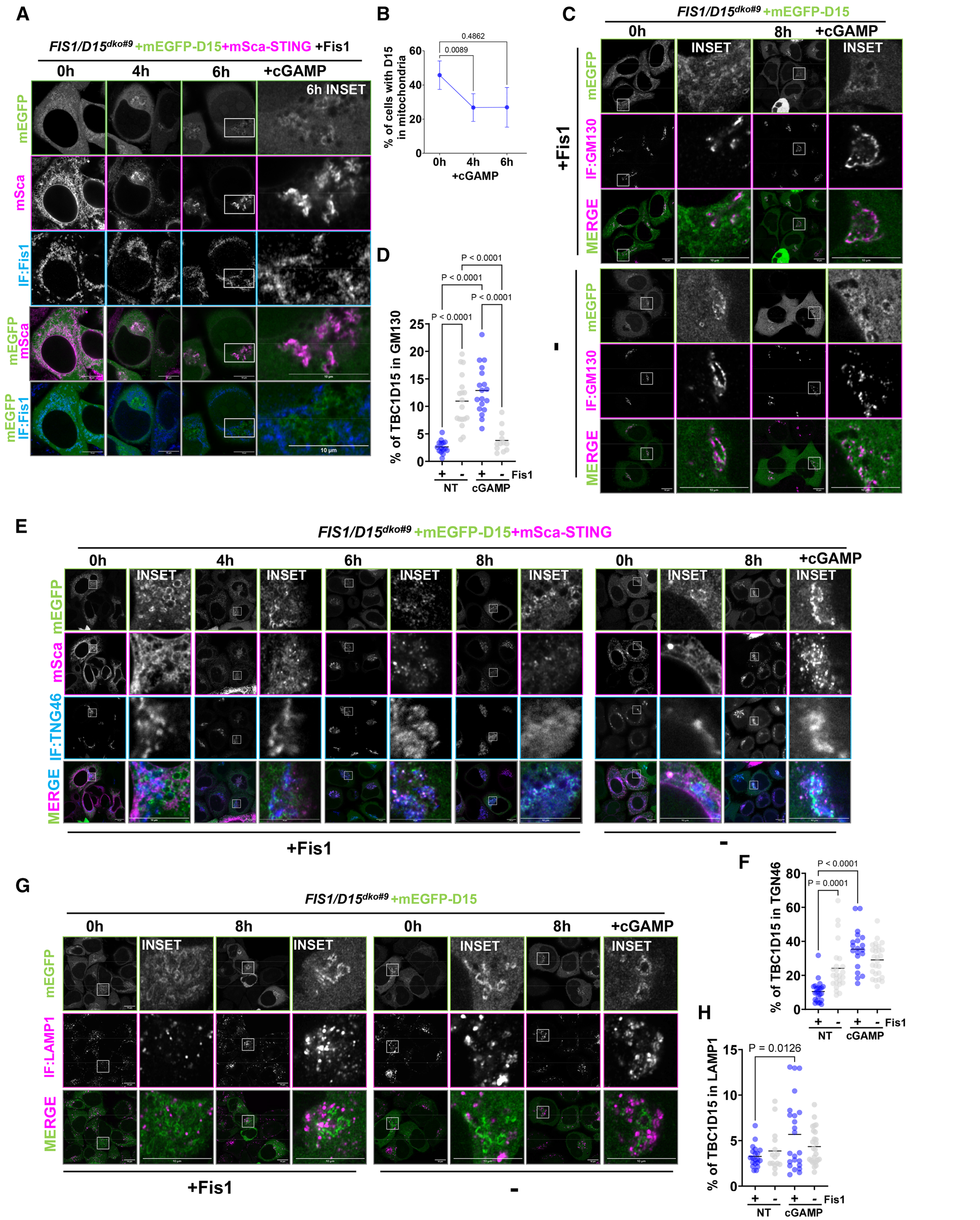

Figure 5. TBC1D15 subcellular localization during STING activation in HeLa cells

(A) Subcellular localization of TBC1D15, STING, and Fis1 followed by confocal microscopy in *FIS1/TBC1D15*$^{dko\#9}$ cells stably expressing mSca-STING in which Fis1 and mEGFP-TBC1D15 were reintroduced. Cells were treated with cGAMP for the specified times (h). Fis1 was detected by immunofluorescence.

(B) Percentage of cells in which mEGFP-TBC1D15 signal was detected at the mitochondria as in (A) ($n = 3$, mean ± SD, one-way ANOVA, Bonferroni, P = p value).

(C) Colocalization of mEGFP-TBC1D15 stably expressed in *FIS1/TBC1D15*$^{dko\#9}$ with *cis*-Golgi marker GM130 in the presence (+) or absence (–) of Fis1 expression. Cells were treated with cGAMP or left with no treatment (NT).

(legend continued on next page)

that TBC1D15 is regulating Rab7A during the late-stages of STING trafficking, as it does during endosomal traffic, by stimulating its GTPase activity[13,14] and possibly by triggering its inactivation and removal during endolysosome maturation.[60] However, we uncovered an additional regulatory layer in which the mitochondria play a role in Rab7A-mediated traffic. The recruitment of TBC1D15 to the mitochondria before it localizes to the Golgi is required to increase its affinity for Rab7A. In turn, a high affinity of TBC1D15 for Rab7A is required during processes that require an increased demand for Rab7A cycling, such as the late stages of activated STING trafficking and proper maturation of endolysosomal vesicles.

Lysosomal STING degradation could be impaired in the absence of Fis1 due to decreased untethering of mitochondria-lysosome and lysosome-lysosome contacts, as observed when a mutant form of Fis1 deficient in recruiting TBC1D15 to the mitochondria is expressed, causing lysosomal clustering and decreased lysosomal-loading of dextran.[16] However, delayed degradation of active STING inside the lysosomes would not maintain the activation of downstream TBK1-IRF3 signaling as we observed.

The role of TBC1D15 on endosomal-GLUT4 glucose transporter translocation,[61] together with our data, suggests that TBC1D15 affinity for Rab7A could adjust the rate of Rab7A-dependent trafficking processes. Based on our data, we propose that recruitment of TBC1D15 to the mitochondria by Fis1 is required to increase its affinity for Rab7A, possibly by modulating posttranslational modifications in TBC1D15. TBC1D1 and TBC1D4 Rab-GAP recruitment to GLUT4 storage vesicles is regulated by phosphorylation, which adjusts their affinity for client Rab-GTPases, representing a mechanism to tune their GTPase activity.[62–68] In conclusion, our study uncovers a mechanism by which mitochondria influence STING signaling through Fis1 and its interactor, the Rab-GAP TBC1D15, and how these proteins could influence inflammation. Our findings highlight the importance of mitochondrial regulation of immune signaling and suggest that targeting the Fis1-TBC1D15-Rab7A axis could offer therapeutic potential for conditions associated with dysregulated STING activity.

Limitations of the study

While we have demonstrated that the absence of Fis1 increases STING stability due to impaired TBC1D15 interaction with Rab7A, which hinders its GTPase activity in HeLa cells and we corroborated the Fis1-dependent STING stabilization phenotype in HCT116 cells, future studies in additional cell lines, particularly primary immune cells and *in vivo* models will be necessary to determine the full generalizability of the Fis1-TBC1D15-Rab7A axis in regulating STING trafficking and innate immune signaling across different physiological contexts. In addition, the precise subcellular spatial location where TBC1D15 acts on Rab7A during STING late-stage trafficking remains to be determined together with the specific downstream mechanisms by which impaired Rab7A cycling leads to STING stabilization.

Further research will shed light on the mechanisms regulating TBC1D15, including its possible posttranslational modifications, such as phosphorylation. In addition, further research on the mechanisms influencing subcellular localization of TBC1D15 and its dynamics, will help elucidate not only the role of Fis1 on the regulation of TBC1D15 but also the mechanisms by which TBC1D15 is recruited to other cellular compartments such as the Golgi and damaged lysosomes.[69]

RESOURCE AVAILABILITY

Lead contact

Requests for resources and reagents should be directed to and will be fulfilled by the lead contact, Richard J. Youle (NINDS, NIH, youler@ninds.nih.gov).

Materials availability

All materials generated in this study are available from the lead contact upon request with a complete materials transfer agreement.

Data and code availability

- The data used to generate the figures and analysis in this paper is available from the lead contact upon request.
- This paper does not report original code.
- Any additional information required to reanalyze the data reported in this paper is available from the lead contact upon request.

ACKNOWLEDGMENTS

The authors thank Dr. Mariusz Karbowski (University of Maryland) for sharing the HeLa *DRP1*^{ko} cells, Dr. Tara D. Fischer for the pHAGE-mEGFP-STING and pHAGE-mSca-STING vectors, and Dr. Shireen A. Sarraf for the pHAGE-mCherry-Rab7A vector. We would also like to thank all other members of the Youle lab for discussions and advice. FACS sorting was performed at the NINDS facility under the supervision of Dr. Dragan Maric. Funding was provided by the Pew Charitable Trusts, through a Pew Latin American Fellowship to E.D.-M. (A-13415) and by the Intramural Program of the National Institute of Neurological Disorders and Stroke to E.D.-M and R.J.Y.

AUTHOR CONTRIBUTIONS

Conceptualization, E.D.-M. and R.J.Y.; methodology, E.D.-M., L.L., J.D.P., and P.-P.Z.; validation, E.D.-M., L.L., and K.R.T.; formal analysis, E.D.-M.; investigation, E.D.-M., L.L., J.D.P., P.-P.Z., and K.R.T.; data curation, E.D.M.; writing – original draft, E.D.-M.; writing – review and editing, E.D.-M., L.L., J.D.P., P.-P.Z., K.R.T., and R.J.Y.; visualization, E.D.-M.; supervision, R.J.Y.; project administration, E.D.-M.; funding acquisition, E.D.-M. and R.J.Y.

(D) Quantifications of TBC1D15 signal within GM130 in (C) (*n* = 12, 18, 18, 12 condition, mean ± SD, one-way ANOVA, Bonferroni, P = *p* value).
(E) Representative images of mEGFP-TBC1D15 and mSca-STING colocalization with TGN46 in the presence (+) or absence (−) of Fis1 expression. Cells were treated with cGAMP for the indicated times (h).
(F) Quantifications of TBC1D15 signal within TGN46 after 8 h of cGAMP treatment in *FIS1/TBC1D15*$^{dko\#9}$+mEGFP-TBC1D15 with (+) or without Fis1 expression (−) in (E) (*n* = 23, 25, 20, 27 cells, mean ± SD, one-way ANOVA, Bonferroni, P = *p* value).
(G) Colocalization of mEGFP-TBC1D15 with LAMP1-positive lysosomes with Fis1 expression (+) or without it (−).
(H) Quantifications of TBC1D15 signal within LAMP1 after 8 h of cGAMP treatment in *FIS1/TBC1D15*$^{dko\#9}$+mEGFP-TBC1D15 with (+) or without Fis1 expression (−) in (G) (*n* = 20, 15, 23, 25 cells, mean ± SD, one-way ANOVA, Bonferroni, P = *p* value). To ease visualization of the confocal imaging, the contrast was adjusted to a different extent in each panel of the figure. For all images the scale bars: 10 μm. *TBC1D15*$^{ko\#5}$ = *D15*$^{ko\#5}$.

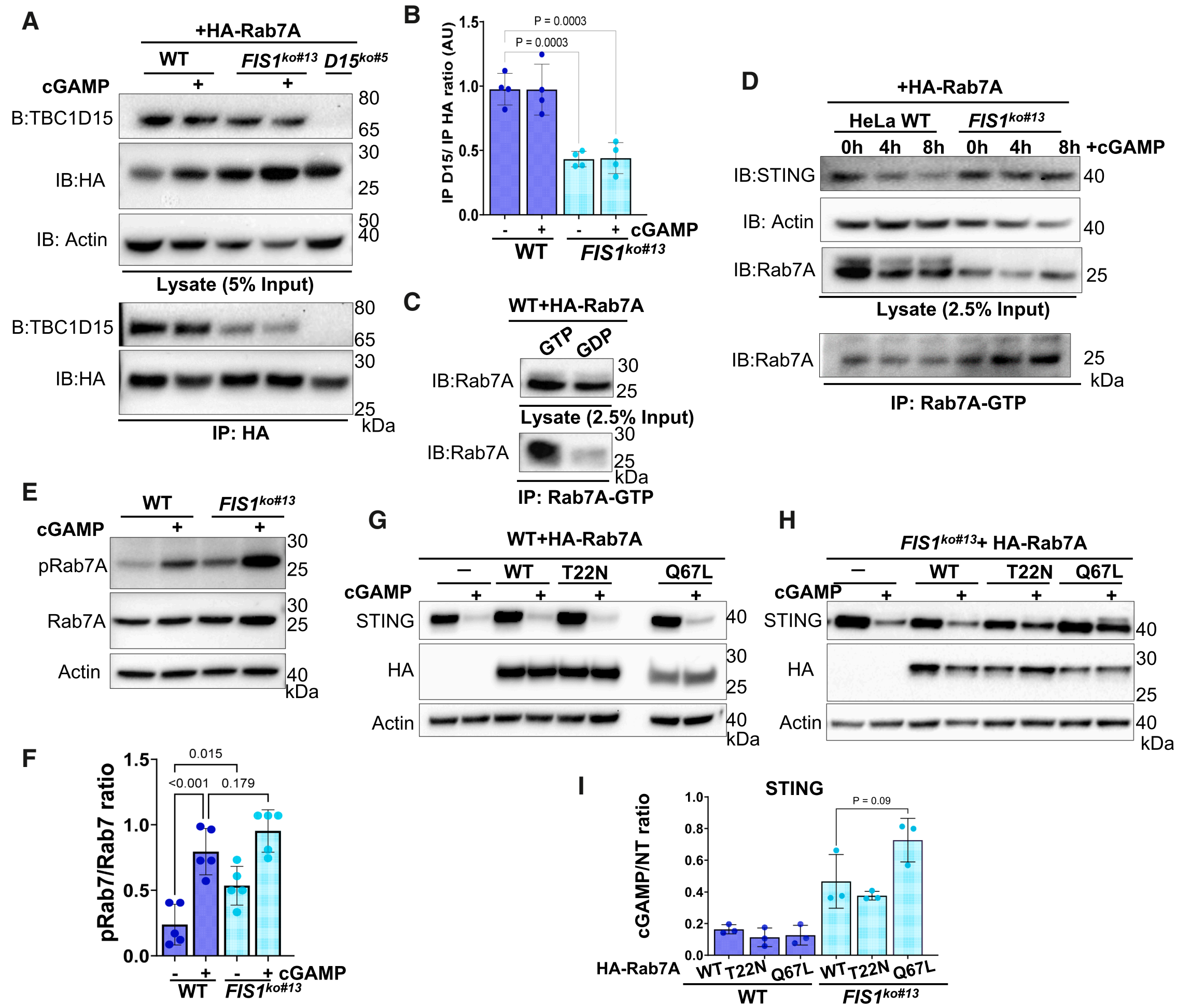

Figure 6. Fis1 absence decreases the interaction between Rab7A and TBC1D15, disturbing the GTP-bound Rab7A equilibrium

(A) HA-Rab7A immunoprecipitated from WT + HA-Rab7A, $FIS1^{ko\#13}$+HA-Rab7A, or $TBC1D15^{ko\#5}$+HA-Rab7A cells with or without cGAMP treatment. TBC1D15 and HA-Rab7A in the lysates or in the eluted fraction were detected by immunoblot. Actin was included as loading control in the total lysate analysis.

(B) Quantifications of IP TBC1D15/IP HA in experiments as in (A) (n = 4, mean ± SD, one-way ANOVA, Bonferroni, P = p value). $TBC1D15^{ko\#5}$ = $D15^{ko\#5}$.

(C) Rab7A immunoblots of eluted HA-Rab7A from WT total cell lysates loaded with GTPγS or GDP and then immunoprecipitated with an anti-Rab7-GTP antibody.

(D) Immunoprecipitation of GTP-bound Rab7A in lysates from WT + HA-Rab7A or $FIS1^{ko\#13}$+HA-Rab7A cells treated for the specified time with cGAMP. Rab7A was immunoblotted in the eluted fraction and total lysates. STING and Actin from total lysates were also assayed.

(E) Rab7A phosphorylation determined by immunoblotting lysates from WT and $FIS1^{ko\#13}$ cells left untreated or after a cGAMP treatment. Total Rab7A and actin were also immunodetected.

(F) Densitometric quantifications, to determine the ratio of pRab7A to Rab7A levels (NT, no treatment; AU, arbitrary units, n = 3, mean ± SD, one-way ANOVA, Bonferroni, P = p value).

(G and H) WT or (H) $FIS1^{ko\#13}$ cells stably expressing HA-Rab7A WT, T22N, or Q67L, treated with or without cGAMP. STING levels were assayed, HA-Rab7 constructs expression was verified by blotting against HA and actin was included as a loading control.

(I) Plot of densitometries to determine STING protein levels as a ratio of cGAMP/NT of experiments as in (G) and (H) (n = 3, mean ± SD, one-way ANOVA, Bonferroni, P = p value).

DECLARATION OF INTERESTS

The authors declare no competing interests.

STAR★METHODS

Detailed methods are provided in the online version of this paper and include the following:

- KEY RESOURCES TABLE
- EXPERIMENTAL MODEL AND STUDY PARTICIPANT DETAILS
  - Human and bacterial cell culture
- METHOD DETAILS
  - Generation of CRISPR gene knockout cell lines
  - Cloning and generation of stably cell line generation
  - Cell treatments
  - 2′3′-cGAMP ELISA
  - Immunoblot assays
  - Immunoprecipitation experiments
  - Immunofluorescence microscopy

- ○ Transmission electron microscopy
- ○ Quantitative real-time PCR
- ● QUANTIFICATION AND STATISTICAL ANALYSIS

SUPPLEMENTAL INFORMATION

Supplemental information can be found online at https://doi.org/10.1016/j.celrep.2026.117994.

Received: March 15, 2025
Revised: April 30, 2026
Accepted: August 26, 2026
Published: September 9, 2026

REFERENCES

1. Mozdy, A.D., McCaffery, J.M., and Shaw, J.M. (2000). Dnm1p GTPase-mediated mitochondrial fission is a multi-step process requiring the novel integral membrane component Fis1p. J. Cell Biol. *151*, 367–380. https://doi.org/10.1083/jcb.151.2.367.
2. Otera, H., Wang, C., Cleland, M.M., Setoguchi, K., Yokota, S., Youle, R.J., and Mihara, K. (2010). Mff is an essential factor for mitochondrial recruitment of Drp1 during mitochondrial fission in mammalian cells. J. Cell Biol. *191*, 1141–1158. https://doi.org/10.1083/jcb.201007152.
3. Koirala, S., Guo, Q., Kalia, R., Bui, H.T., Eckert, D.M., Frost, A., and Shaw, J.M. (2013). Interchangeable adaptors regulate mitochondrial dynamin assembly for membrane scission. Proc. Natl. Acad. Sci. USA *110*, E1342–E1351. https://doi.org/10.1073/pnas.1300855110.
4. Osellame, L.D., Singh, A.P., Stroud, D.A., Palmer, C.S., Stojanovski, D., Ramachandran, R., and Ryan, M.T. (2016). Cooperative and independent roles of the Drp1 adaptors Mff, MiD49 and MiD51 in mitochondrial fission. J. Cell Sci. *129*, 2170–2181. https://doi.org/10.1242/jcs.185165.
5. Losón, O.C., Song, Z., Chen, H., and Chan, D.C. (2013). Fis1, Mff, MiD49, and MiD51 mediate Drp1 recruitment in mitochondrial fission. Mol. Biol. Cell *24*, 659–667. https://doi.org/10.1091/mbc.E12-10-0721.
6. Shen, Q., Yamano, K., Head, B.P., Kawajiri, S., Cheung, J.T.M., Wang, C., Cho, J.-H., Hattori, N., Youle, R.J., and van der Bliek, A.M. (2014). Mutations in Fis1 disrupt orderly disposal of defective mitochondria. Mol. Biol. Cell *25*, 145–159. https://doi.org/10.1091/mbc.E13-09-0525.
7. Yamano, K., Fogel, A.I., Wang, C., van der Bliek, A.M., and Youle, R.J. (2014). Mitochondrial Rab GAPs govern autophagosome biogenesis during mitophagy. eLife *3*, e01612. https://doi.org/10.7554/eLife.01612.
8. Kleele, T., Rey, T., Winter, J., Zaganelli, S., Mahecic, D., Perreten Lambert, H., Ruberto, F.P., Nemir, M., Wai, T., Pedrazzini, T., and Manley, S. (2021). Distinct fission signatures predict mitochondrial degradation or biogenesis. Nature *593*, 435–439. https://doi.org/10.1038/s41586-021-03510-6.
9. Tábara, L.C., Burr, S.P., Frison, M., Chowdhury, S.R., Paupe, V., Nie, Y., Johnson, M., Villar-Azpillaga, J., Viegas, F., Segawa, M., et al. (2024). MTFP1 controls mitochondrial fusion to regulate inner membrane quality control and maintain mtDNA levels. Cell *187*, 3619–3637.e27. https://doi.org/10.1016/j.cell.2024.05.017.
10. Zhang, Z., Sliter, D.A., Bleck, C.K.E., and Ding, S. (2019). Fis1 deficiencies differentially affect mitochondrial quality in skeletal muscle. Mitochondrion *49*, 217–226. https://doi.org/10.1016/j.mito.2019.09.005.
11. Dickinson, M.E., Flenniken, A.M., Ji, X., Teboul, L., Wong, M.D., White, J.K., Meehan, T.F., Weninger, W.J., Westerberg, H., Adissu, H., et al. (2016). High-throughput discovery of novel developmental phenotypes. Nature *537*, 508–514. https://doi.org/10.1038/nature19356.
12. Onoue, K., Jofuku, A., Ban-Ishihara, R., Ishihara, T., Maeda, M., Koshiba, T., Itoh, T., Fukuda, M., Otera, H., Oka, T., et al. (2013). Fis1 acts as a mitochondrial recruitment factor for TBC1D15 that is involved in regulation of mitochondrial morphology. J. Cell Sci. *126*, 176–185. https://doi.org/10.1242/jcs.111211.
13. Zhang, X.-M., Walsh, B., Mitchell, C.A., and Rowe, T. (2005). TBC domain family, member 15 is a novel mammalian Rab GTPase-activating protein with substrate preference for Rab7. Biochem. Biophys. Res. Commun. *335*, 154–161. https://doi.org/10.1016/j.bbrc.2005.07.070.
14. Peralta, E.R., Martin, B.C., and Edinger, A.L. (2010). Differential effects of TBC1D15 and mammalian Vps39 on Rab7 activation state, lysosomal morphology, and growth factor dependence. J. Biol. Chem. *285*, 16814–16821. https://doi.org/10.1074/jbc.M110.111633.
15. Wong, Y.C., Ysselstein, D., and Krainc, D. (2018). Mitochondria-lysosome contacts regulate mitochondrial fission via RAB7 GTP hydrolysis. Nature *554*, 382–386. https://doi.org/10.1038/nature25486.
16. Wong, Y.C., Kim, S., Cisneros, J., Molakal, C.G., Song, P., Lubbe, S.J., and Krainc, D. (2022). Mid51/Fis1 mitochondrial oligomerization complex drives lysosomal untethering and network dynamics. J. Cell Biol. *221*, e202206140. https://doi.org/10.1083/jcb.202206140.
17. Liou, Y.-H., Personnaz, J., Jacobi, D., Knudsen, N.H., Chalom, M.M., Starost, K.A., Nnah, I.C., and Lee, C.-H. (2022). Hepatic Fis1 regulates mitochondrial integrated stress response and improves metabolic homeostasis. JCI Insight *7*, e150041. https://doi.org/10.1172/jci.insight.150041.
18. Luteijn, R.D., Zaver, S.A., Gowen, B.G., Wyman, S.K., Garelis, N.E., Onia, L., McWhirter, S.M., Katibah, G.E., Corn, J.E., Woodward, J.J., and Raulet, D.H. (2019). SLC19A1 transports immunoreactive cyclic dinucleotides. Nature *573*, 434–438. https://doi.org/10.1038/s41586-019-1553-0.
19. Ishikawa, H., and Barber, G.N. (2008). STING is an endoplasmic reticulum adaptor that facilitates innate immune signalling. Nature *455*, 674–678. https://doi.org/10.1038/nature07317.
20. Saitoh, T., Fujita, N., Hayashi, T., Takahara, K., Satoh, T., Lee, H., Matsunaga, K., Kageyama, S., Omori, H., Noda, T., et al. (2009). Atg9a controls dsDNA-driven dynamic translocation of STING and the innate immune response. Proc. Natl. Acad. Sci. USA *106*, 20842–20846. https://doi.org/10.1073/pnas.0911267106.
21. Ishikawa, H., Ma, Z., and Barber, G.N. (2009). STING regulates intracellular DNA-mediated, type I interferon-dependent innate immunity. Nature *461*, 788–792. https://doi.org/10.1038/nature08476.
22. Shu, C., Yi, G., Watts, T., Kao, C.C., and Li, P. (2012). Structure of STING bound to cyclic di-GMP reveals the mechanism of cyclic dinucleotide recognition by the immune system. Nat. Struct. Mol. Biol. *19*, 722–724. https://doi.org/10.1038/nsmb.2331.
23. Konno, H., Konno, K., and Barber, G.N. (2013). Cyclic dinucleotides trigger ULK1 (ATG1) phosphorylation of STING to prevent sustained innate immune signaling. Cell *155*, 688–698. https://doi.org/10.1016/j.cell.2013.09.049.
24. Sun, L., Wu, J., Du, F., Chen, X., and Chen, Z.J. (2013). Cyclic GMP-AMP synthase is a cytosolic DNA sensor that activates the type I interferon pathway. Science *339*, 786–791. https://doi.org/10.1126/science.1232458.
25. Wu, J., Sun, L., Chen, X., Du, F., Shi, H., Chen, C., and Chen, Z.J. (2013). Cyclic GMP-AMP Is an Endogenous Second Messenger in Innate Immune Signaling by Cytosolic DNA Jiaxi. Science, 826–831.
26. Tanaka, Y., and Chen, Z.J. (2012). STING specifies IRF3 phosphorylation by TBK1 in the cytosolic DNA signaling pathway. Sci. Signal. *5*, ra20. https://doi.org/10.1126/scisignal.2002521.
27. Kemmoku, H., Kuchitsu, Y., Mukai, K., and Taguchi, T. (2022). Specific association of TBK1 with the trans-Golgi network following STING stimulation. Cell Struct. Funct. *47*, 19–30. https://doi.org/10.1247/csf.21080.
28. Gonugunta, V.K., Sakai, T., Pokatayev, V., Yang, K., Wu, J., Dobbs, N., and Yan, N. (2017). Trafficking-Mediated STING Degradation Requires Sorting to Acidified Endolysosomes and Can Be Targeted to Enhance Anti-tumor Response. Cell Rep. *21*, 3234–3242. https://doi.org/10.1016/j.celrep.2017.11.061.
29. Gentili, M., Liu, B., Papanastasiou, M., Dele-Oni, D., Schwartz, M.A., Carlson, R.J., Al'Khafaji, A.M., Krug, K., Brown, A., Doench, J.G., et al.

(2023). ESCRT-dependent STING degradation inhibits steady-state and cGAMP-induced signalling. Nat. Commun. *14*, 611. https://doi.org/10.1038/s41467-023-36132-9.

30. Balka, K.R., Venkatraman, R., Saunders, T.L., Shoppee, A., Pang, E.S., Magill, Z., Homman-Ludiye, J., Huang, C., Lane, R.M., York, H.M., et al. (2023). Termination of STING responses is mediated via ESCRT-dependent degradation. EMBO J. *42*, e112712. https://doi.org/10.15252/embj.2022112712.
31. Kuchitsu, Y., Mukai, K., Uematsu, R., Takaada, Y., Shinojima, A., Shindo, R., Shoji, T., Hamano, S., Ogawa, E., Sato, R., et al. (2023). STING signalling is terminated through ESCRT-dependent microautophagy of vesicles originating from recycling endosomes. Nat. Cell Biol. *25*, 453–466. https://doi.org/10.1038/s41556-023-01098-9.
32. Pokatayev, V., Yang, K., Tu, X., Dobbs, N., Wu, J., Kalb, R.G., and Yan, N. (2020). Homeostatic regulation of STING protein at the resting state by stabilizer TOLLIP. Nat. Immunol. *21*, 158–167. https://doi.org/10.1038/s41590-019-0569-9.
33. Gui, X., Yang, H., Li, T., Tan, X., Shi, P., Li, M., Du, F., and Chen, Z.J. (2019). Autophagy induction via STING trafficking is a primordial function of the cGAS pathway. Nature *567*, 262–266. https://doi.org/10.1038/s41586-019-1006-9.
34. Muñoz-Braceras, S., and Escalante, R. (2016). Analysis of Relevant Parameters for Autophagic Flux Using HeLa Cells Expressing EGFP-LC3. Methods Mol. Biol. *1449*, 313–329. https://doi.org/10.1007/978-1-4939-3756-1_20.
35. Jofuku, A., Ishihara, N., and Mihara, K. (2005). Analysis of functional domains of rat mitochondrial Fis1, the mitochondrial fission-stimulating protein. Biochem. Biophys. Res. Commun. *333*, 650–659. https://doi.org/10.1016/j.bbrc.2005.05.154.
36. Ihenacho, U.K., Toro, R., Mansour, R.H., and Hill, R.B. (2023). A conserved, noncanonical insert in FIS1 mediates TBC1D15 and DRP1 recruitment for mitochondrial fission. J. Biol. Chem. *299*, 105303. https://doi.org/10.1016/j.jbc.2023.105303.
37. Modica, G., and Lefrancois, S. (2020). Post-translational modifications: How to modulate Rab7 functions. Small GTPases *11*, 167–173. https://doi.org/10.1080/21541248.2017.1387686.
38. Shinde, S.R., and Maddika, S. (2016). PTEN modulates EGFR late endocytic trafficking and degradation by dephosphorylating Rab7. Nat. Commun. *7*, 10689. https://doi.org/10.1038/ncomms10689.
39. Talaia, G., Bentley-DeSousa, A., and Ferguson, S.M. (2024). Lysosomal TBK1 responds to amino acid availability to relieve Rab7-dependent mTORC1 inhibition. EMBO J. *43*, 3948–3967. https://doi.org/10.1038/s44318-024-00180-8.
40. Ritter, J.L., Zhu, Z., Thai, T.C., Mahadevan, N.R., Mertins, P., Knelson, E.H., Piel, B.P., Han, S., Jaffe, J.D., Carr, S.A., et al. (2020). Phosphorylation of RAB7 by TBK1/IKKε Regulates Innate Immune Signaling in Triple-Negative Breast Cancer. Cancer Res. *80*, 44–56. https://doi.org/10.1158/0008-5472.CAN-19-1310.
41. Spinosa, M.R., Progida, C., De Luca, A., Colucci, A.M.R., Alifano, P., and Bucci, C. (2008). Functional characterization of Rab7 mutant proteins associated with Charcot-Marie-Tooth type 2B disease. J. Neurosci. *28*, 1640–1648. https://doi.org/10.1523/JNEUROSCI.3677-07.2008.
42. McCray, B.A., Skordalakes, E., and Taylor, J.P. (2010). Disease mutations in Rab7 result in unregulated nucleotide exchange and inappropriate activation. Hum. Mol. Genet. *19*, 1033–1047. https://doi.org/10.1093/hmg/ddp567.
43. Marques, E., Kramer, R., and Ryan, D.G. (2024). Multifaceted mitochondria in innate immunity. NPJ Metab. Health Dis. *2*, 6. https://doi.org/10.1038/s44324-024-00008-3.
44. Sabesin, S.M., and Frase, S. (1977). Electron microscopic studies of the assembly, intracellular transport, and secretion of chylomicrons by rat intestine. J. Lipid Res. *18*, 496–511.
45. Bonfanti, L., Mironov, A.A., Martínez-Menárguez, J.A., Martella, O., Fusella, A., Baldassarre, M., Buccione, R., Geuze, H.J., Mironov, A.A., and Luini, A. (1998). Procollagen Traverses the Golgi Stack without Leaving the Lumen of Cisternae. Cell *95*, 993–1003. https://doi.org/10.1016/S0092-8674(00)81723-7.
46. Ellinger, A., and Pavelka, M. (1984). Effect of monensin on the Golgi apparatus of absorptive cells in the small intestine of the rat. Morphological and cytochemical studies. Cell Tissue Res. *235*, 187–194. https://doi.org/10.1007/BF00213739.
47. Zhang, G.F., Driouich, A., and Staehelin, L.A. (1993). Effect of monensin on plant Golgi: re-examination of the monensin-induced changes in cisternal architecture and functional activities of the Golgi apparatus of sycamore suspension-cultured cells. J. Cell Sci. *104*, 819–831. https://doi.org/10.1242/jcs.104.3.819.
48. Vanneste, M., Huang, Q., Li, M., Moose, D., Zhao, L., Stamnes, M.A., Schultz, M., Wu, M., and Henry, M.D. (2019). High content screening identifies monensin as an EMT-selective cytotoxic compound. Sci. Rep. *9*, 1200. https://doi.org/10.1038/s41598-018-38019-y.
49. Sakaguchi, T., Leser, G.P., and Lamb, R.A. (1996). The ion channel activity of the influenza virus M2 protein affects transport through the Golgi apparatus. J. Cell Biol. *133*, 733–747. https://doi.org/10.1083/jcb.133.4.733.
50. Saraste, J., and Prydz, K. (2021). Assembly and Cellular Exit of Coronaviruses: Hijacking an Unconventional Secretory Pathway from the Pre-Golgi Intermediate Compartment via the Golgi Ribbon to the Extracellular Space. Cells *10*, 503. https://doi.org/10.3390/cells10030503.
51. Maeda, Y., Ide, T., Koike, M., Uchiyama, Y., and Kinoshita, T. (2008). GPHR is a novel anion channel critical for acidification and functions of the Golgi apparatus. Nat. Cell Biol. *10*, 1135–1145. https://doi.org/10.1038/ncb1773.
52. Shang, G., Zhang, C., Chen, Z.J., Bai, X.-C., and Zhang, X. (2019). Cryo-EM structures of STING reveal its mechanism of activation by cyclic GMP-AMP. Nature *567*, 389–393. https://doi.org/10.1038/s41586-019-0998-5.
53. Liu, B., Carlson, R.J., Pires, I.S., Gentili, M., Feng, E., Hellier, Q., Schwartz, M.A., Blainey, P.C., Irvine, D.J., and Hacohen, N. (2023). Human STING is a proton channel. Science *381*, 508–514. https://doi.org/10.1126/science.adf8974.
54. Xun, J., Zhang, Z., Lv, B., Lu, D., Yang, H., Shang, G., and Tan, J.X. (2024). A conserved ion channel function of STING mediates noncanonical autophagy and cell death. EMBO Rep. *25*, 544–569. https://doi.org/10.1038/s44319-023-00045-x.
55. Suklabaidya, S., Mohanty, S., Reider, I.E., White, J., Colter, D., McCormick, S.M., Shembade, N., Choi, Y.B., Norbury, C.C., and Harhaj, E.W. (2026). Negative feedback regulation of STING signaling by TAX1BP1-directed Golgiphagy. Nat. Commun. *17*, 2762. https://doi.org/10.1038/s41467-026-69422-z.
56. Tu, X., Chu, T.-T., Jeltema, D., Abbott, K., Yang, K., Xing, C., Han, J., Dobbs, N., and Yan, N. (2022). Interruption of post-Golgi STING trafficking activates tonic interferon signaling. Nat. Commun. *13*, 6977. https://doi.org/10.1038/s41467-022-33765-0.
57. Zhu, H., Zhang, R., Yi, L., Tang, Y.-D., and Zheng, C. (2022). UNC93B1 attenuates the cGAS-STING signaling pathway by targeting STING for autophagy-lysosome degradation. J. Med. Virol. *94*, 4490–4501. https://doi.org/10.1002/jmv.27860.
58. Pan, M., Yin, Y., Hu, T., Wang, X., Jia, T., Sun, J., Wang, Q., Meng, W., Zhu, J., Dai, C., et al. (2023). UXT attenuates the CGAS-STING1 signaling by targeting STING1 for autophagic degradation. Autophagy *19*, 440–456. https://doi.org/10.1080/15548627.2022.2076192.
59. Shoji, T., Shinojima, A., Kusumi, S., Koga, D., Mukai, K., Nakayama, J., Higashiyama, S., Kuchitsu, Y., and Taguchi, T. (2024). A PI(3,5)P2/ESCRT-III axis terminates STING signalling by facilitating TSG101-mediated lysosomal microautophagy. bioRxiv. https://doi.org/10.1101/2024.05.26.595979.

60. Jongsma, M.L., Bakker, J., Cabukusta, B., Liv, N., van Elsland, D., Fermie, J., Akkermans, J.L., Kuijl, C., van der Zanden, S.Y., Janssen, L., et al. (2020). SKIP-HOPS recruits TBC1D15 for a Rab7-to-Arl8b identity switch to control late endosome transport. EMBO J. *39*, e102301. https://doi.org/10.15252/embj.2019102301.

61. Wu, J., Cheng, D., Liu, L., Lv, Z., and Liu, K. (2019). TBC1D15 affects glucose uptake by regulating GLUT4 translocation. Gene *683*, 210–215. https://doi.org/10.1016/j.gene.2018.10.025.

62. Kane, S., Sano, H., Liu, S.C.H., Asara, J.M., Lane, W.S., Garner, C.C., and Lienhard, G.E. (2002). A method to identify serine kinase substrates. Akt phosphorylates a novel adipocyte protein with a Rab GTPase-activating protein (GAP) domain. J. Biol. Chem. *277*, 22115–22118. https://doi.org/10.1074/jbc.C200198200.

63. Sano, H., Kane, S., Sano, E., Mîinea, C.P., Asara, J.M., Lane, W.S., Garner, C.W., and Lienhard, G.E. (2003). Insulin-stimulated phosphorylation of a Rab GTPase-activating protein regulates GLUT4 translocation. J. Biol. Chem. *278*, 14599–14602. https://doi.org/10.1074/jbc.C300063200.

64. Taylor, E.B., An, D., Kramer, H.F., Yu, H., Fujii, N.L., Roeckl, K.S.C., Bowles, N., Hirshman, M.F., Xie, J., Feener, E.P., and Goodyear, L.J. (2008). Discovery of TBC1D1 as an insulin-AICAR-and contraction-stimulated signaling nexus in mouse skeletal muscle. J. Biol. Chem. *283*, 9787–9796. https://doi.org/10.1074/jbc.M708839200.

65. Pehmøller, C., Treebak, J.T., Birk, J.B., Chen, S., Mackintosh, C., Hardie, D.G., Richter, E.A., and Wojtaszewski, J.F.P. (2009). Genetic disruption of AMPK signaling abolishes both contraction- and insulin-stimulated TBC1D1 phosphorylation and 14-3-3 binding in mouse skeletal muscle. Am. J. Physiol. Endocrinol. Metab. *297*, E665–E675. https://doi.org/10.1152/ajpendo.00115.2009.

66. Eickelschulte, S., Hartwig, S., Leiser, B., Lehr, S., Joschko, V., Chokkalingam, M., Chadt, A., and Al-Hasani, H. (2021). AKT/AMPK-mediated phosphorylation of TBC1D4 disrupts the interaction with insulin-regulated aminopeptidase. J. Biol. Chem. *296*, 100637. https://doi.org/10.1016/j.jbc.2021.100637.

67. Chen, Y., Wang, Y., Zhang, J., Deng, Y., Jiang, L., Song, E., Wu, X.S., Hammer, J.A., Xu, T., and Lippincott-Schwartz, J. (2012). Rab10 and myosin-Va mediate insulin-stimulated GLUT4 storage vesicle translocation in adipocytes. J. Cell Biol. *198*, 545–560. https://doi.org/10.1083/jcb.201111091.

68. Li, D.T., Habtemichael, E.N., Julca, O., Sales, C.I., Westergaard, X.O., DeVries, S.G., Ruiz, D., Sayal, B., and Bogan, J.S. (2019). GLUT4 storage vesicles: specialized organelles for regulated trafficking. Yale J. Biol. Med. *92*, 453–470.

69. Bhattacharya, A., Mukherjee, R., Kuncha, S.K., Brunstein, M.E., Rathore, R., Junek, S., Münch, C., and Dikic, I. (2023). A lysosome membrane regeneration pathway depends on TBC1D15 and autophagic lysosomal reformation proteins. Nat. Cell Biol. *25*, 685–698. https://doi.org/10.1038/s41556-023-01125-9.

70. Karbowski, M., Neutzner, A., and Youle, R.J. (2007). The mitochondrial E3 ubiquitin ligase MARCH5 is required for Drp1 dependent mitochondrial division. J. Cell Biol. *178*, 71–84. https://doi.org/10.1083/jcb.200611064.

71. Wang, C. (2020). A Sensitive and Quantitative mKeima Assay for Mitophagy via FACS. Curr. Protoc. Cell Biol. *86*, e99. https://doi.org/10.1002/cpcb.99.

72. Fang, R., Wang, C., Jiang, Q., Lv, M., Gao, P., Yu, X., Mu, P., Zhang, R., Bi, S., Feng, J.-M., and Jiang, Z. (2017). NEMO-IKKβ Are Essential for IRF3 and NF-κB Activation in the cGAS-STING Pathway. J. Immunol. *199*, 3222–3233. https://doi.org/10.4049/jimmunol.1700699.

73. Wu, X., and Hammer, J.A. (2021). ZEISS Airyscan: Optimizing Usage for Fast, Gentle, Super-Resolution Imaging. Methods Mol. Biol. *2304*, 111–130. https://doi.org/10.1007/978-1-0716-1402-0_5.

74. Schindelin, J., Arganda-Carreras, I., Frise, E., Kaynig, V., Longair, M., Pietzsch, T., Preibisch, S., Rueden, C., Saalfeld, S., Schmid, B., et al. (2012). Fiji: an open-source platform for biological-image analysis. Nat. Methods *9*, 676–682. https://doi.org/10.1038/nmeth.2019.

75. Zhang, Q., Lee, W.-C.A., Paul, D.L., and Ginty, D.D. (2019). Multiplexed peroxidase-based electron microscopy labeling enables simultaneous visualization of multiple cell types. Nat. Neurosci. *22*, 828–839. https://doi.org/10.1038/s41593-019-0358-7.

76. Livak, K.J., and Schmittgen, T.D. (2001). Analysis of relative gene expression data using real-time quantitative PCR and the 2(-Delta Delta C(T)) Method. Methods *25*, 402–408. https://doi.org/10.1006/meth.2001.1262.

STAR★METHODS

KEY RESOURCES TABLE

REAGENT or RESOURCE	SOURCE	IDENTIFIER
**Antibodies**		
Actin	Sigma-Aldrich	MAB1501; RRID: AB_2223041
HSP90	Cell Signaling Technology	4877; RRID: AB_2233307
FIS1	Proteintech	10956-1-AP; RRID: AB_2102532
TBC1D15	Abcam	ab121396; RRID: AB_11128143
TBC1D17	Proteintech	20482-1-AP; RRID: AB_11232420
PINK1	Cell Signaling Technology	6946; RRID: AB_11179069
HA	Cell Signaling Technology	3724; RRID: AB_1549585
Rab7	NewEast Biosciences	26923; RRID: AB_2629401
pRab7 (S72)	Abcam	ab302494; RRID: AB_2933985
pSTING (Ser366)	Cell Signaling Technology	50907; RRID: AB_2827656
STING	Cell Signaling Technology	13647; RRID: AB_2732796
pTBK1 (Ser172)	Cell Signaling Technology	5483; RRID: AB_10693472
TBK1	Cell Signaling Technology	3504; RRID: AB_2255663
pIRF3 (S386)	Abcam	ab76493; RRID: AB_1523836
IRF3	Cell Signaling Technology	4302; RRID: AB_1904036
TOMM20	Santa Cruz Biotechnology	Sc-17764; RRID: AB_628381
PDI	Thermo Fisher Scientific	MA3-019; RRID: AB_2163120
GM130	BD Transduction Laboratories	610823; RRID: AB_398142
TGN46	Bio-Rad	AHP500GT; RRID: AB_324049
LAMP1	Cell Signaling Technology	15665; RRID: AB_2798750
HRP-Goat Anti-Rabbit IgG	NewEast Biosciences	29002
Anti-rabbit IgG, HRP-linked	Cell Signaling Technology	7074; RRID: AB_2099233
Amersham ECL Rabbit IgG, HRP-linked $F(ab')_2$ fragment (from donkey)	Cytiva	NA9340; RRID: AB_772191
Amersham ECL Mouse IgG, HRP-linked $F(ab')_2$ fragment (from sheep)	Cytiva	NA9310; RRID: AB_772193
GFP-booster ATTO488	ChromoTek	gba488; RRID: AB_2631386
Alexa Fluor 633 goat anti-mouse IgG (H + L), highly cross-adsorbed	Thermo Fisher Scientific	A-21052; RRID: AB_2535719
Alexa Fluor 405 goat anti-rabbit IgG (H + L), highly cross-adsorbed	Thermo Fisher Scientific	A-31556; RRID: AB_221605
Alex Fluor 488 goat anti-mouse IgG (H + L), highly cross-adsorbed	Thermo Fisher Scientific	A-11029; RRID: AB_2534088
Alexa Fluor 488 goat anti-rabbit IgG (H + L), highly cross-adsorbed	Thermo Fisher Scientific	A-11034; RRID: AB_2576217
Alexa Fluor 546 goat anti-mouse IgG (H + L), highly cross-adsorbed	Thermo Fisher Scientific	A-11030; RRID: AB_2737024
Alexa Fluor 405 donkey anti-sheep IgG (H + L)	Abcam	ab175676; RRID: AB_3754651
**Chemicals, peptides, and recombinant proteins**		
DMEM (Dulbecco's modified eagle medium), high glucose, no glutamine, no sodium pyruvate	Thermo Fisher Scientific	11960044
DMEM (Dulbecco's modified eagle medium), high glucose, no glutamine, no sodium pyruvate, no phenol red	Thermo Fisher Scientific	31053028

(*Continued on next page*)

Continued

REAGENT or RESOURCE	SOURCE	IDENTIFIER
Fetal bovine serum, dialyzed by ultrafiltration against 0.15 NaCl	Sigma-Aldrich	F0392-100ML
FBS certified one shot	Thermo Fisher Scientific	16000-077
Sodium pyruvate (100 mM)	Thermo Fisher Scientific	11360070
GlutaMAX supplement	Thermo Fisher Scientific	35050061
HBSS, no calcium, no magnesium, no phenol red	Thermo Fisher Scientific	14175095
AccGENE 10× PBS	Lonza	51226
TrypLE express enzyme (1×), phenol red (trypsin)	Thermo Fisher Scientific	12605010
2′, 3′-cyclic GMP-AMP Sodium Salt (cGAMP)	ChemieTek	CT-CGAMP
cOmplete^{TM}, Mini, EDTA-free Protease Inhibitor Cocktail	Roche	11836170001
Sodium fluoride	Sigma-Aldrich	S1504
Bambanker	Fujifilm Wako Chemicals	302-14681
TWEEN 20	Sigma-Aldrich	P1379
Skim milk powder	Millipore Sigma	1153630500
Restore PLUS Western Blot Stripping Buffer	Thermo Fisher Scientific	46430
DL-Dithiothreitol	Sigma-Aldrich	D5545
4× LDS Sample Buffer	GenScript	M00676
Paraformaldehyde 16% aqueous solution EM grade	Electron Microscopy Sciences	15710
Triton X-100	Sigma-Aldrich	X100-100ML
DAPI	Thermo Fisher Scientific	62248
ibidi mounting medium	ibidi	50001
Fetal bovine serum, premium select, heat inactivated	R&D Systems	S11595H
EDTA (0.5 M), pH 8.0	Quality Biological, Inc.	351-027-101
LDS	Research Products International	L26200-100.0
Glycerol	Bio Basic	GB0232
Trizma base	Sigma-Aldrich	T4661
Sodium chloride (5 M)	Quality Biological, Inc.	351-036-101
TRIS-HCl	IBI Scientific	IB70162
Tris HCl (1 M), pH 7.5	Quality Biological, Inc.	351-006-101
Hexadimethrine bromide (polybrene)	Sigma-Aldrich	H9268
Puromycin (solution)	Invivogen	ant-pr-1
Polyethylenimine (PEI 25K)	PolySciences	23966
Lipofectamine 2000 Transfection Reagent	Thermo Fisher Scientific	11668027
Poly(dA:dT) naked	Invivogen	tlrl-patn
Antimycin A from *Streptomyces* sp.	Sigma-Aldrich	A8674
Chloroquine	Sigma-Aldrich	C6628
Oligomycin	Calbiochem, Sigma-Aldrich	495455
Fisher BioReagents Bovine Serum Albumin, Heat Shock Treated	Fisher Scientific	BP1600-100
Sodium azide	Sigma-Aldrich	71289
MES SDS Running Buffer Powder	GenScript	M00677
Tris-MOPS-SDS Running Buffer Powder	GenScript	M00138
Luria-Bertani (LB) Broth +1.5% Agar	KD Medical	BLF-7070
Carbenicillin (Disodium)	GoldBio	C-103-100

(*Continued on next page*)

Continued

REAGENT or RESOURCE	SOURCE	IDENTIFIER
2M Magnesium Chloride	Quality Biological, Inc.	340-034-060
Kanamycin sulfate *Streptomyces kanamyceticus*	Sigma-Aldrich	K1377
jetOPTIMUS DNA Transfection Reagent	Polyplus	101000006
Opti-MEM, Reduced Serum Medium	Thermo Fisher Scientific	31985062
M-PER Mammalian Protein Extraction Reagent	Thermo Fisher Scientific	78501
HEPES Buffer Solution (1M)	Thermo Fisher Scientific	15630
Sorensen's Phosphate Buffer	Electron Microscopy Sciences	11601
Ammonium Chloride	Sigma-Aldrich	A-5666
Bovine Serum Albumin	Sigma-Aldrich	A7906
Glutaraldehyde, 10% aqueous	Electron Microscopy Sciences	16120
HQ Silver Enhancement Kit	NanoProbes	2012-45ML
Osmium, 4% aqueous	Electron Microscopy Sciences	19150
Potassium Ferrocyanide	Fisher Scientific	P236-500
Tannic acid	Electron Microscopy Sciences	21700
Uranyl acetate	Electron Microscopy Sciences	22400
Sodium acetate trihydrate	Electron Microscopy Sciences	21120
Ethyl Alcohol, 200 proof	The Warner-Graham Co.	6505001050000
Gelatine capsule, size "0"	Electron Microscopy Sciences	70110
Embed 812 Embedding Kit with Dmp-30	Electron Microscopy Sciences	14120
Lead Citrate Reynold's Stain 3%	Electron Microscopy Sciences	22410
Calcium Chloride	Quality Biological, Inc.	233-140-8
Glycine	Sigma-Aldrich	G7126
Poly-L-lysine hydrobromide	Sigma-Aldrich	P2636
PowerUp SYBR Green Master Mix	Thermo Fisher Scientific	A25742
**Critical commercial assays**		
Amersham ECL Prime Western Blotting Detection Reagent	Cytiva	RPN2236
SuperSignal West FEMTO Maximum Sensitivity Substrate	Thermo Fisher Scientific	34094
pGEM-T Easy Vector System I	Promega	A1360
Pierce BCA Protein Assay	Thermo Fisher Scientific	23227
Quick-RNA MiniPrep Plus kit	Zymo Research	R1058
NEBuilder HiFi DNA Assembly Cloning Kit with competent cells	New England Biolabs	E5520S
Rab7 Pull-Down Activation Assay Kit	NewEast Biosciences	82501
Phusion Hot Start II High-Fidelity PCR Master Mixes	Thermo Fisher Scientific	F565S
2'3'-cGAMP ELISA Kit	Cayman Chemical Company	501700-Solid
Pierce Anti-HA Magnetic Beads	Thermo Fisher Scientific	88836
Trans-Blot Turbo RTA Midi 0.2 μm PVDF Transfer Kit	Bio-Rad	1704273
High-Capacity cDNA Reverse Transcription Kit	Thermo Scientific	4368814
**Experimental models: Cell lines**		
NEB Stable Competent *E. coli*, High Efficiency cells	New England Biolabs	C3040H
NEB 5-alpha Competent *E. coli*	New England Biolabs	C2987H
HEK 293T	ATCC	CRL-3216
HeLa	ATCC	CCL-2.2

(*Continued on next page*)

CellPress
OPEN ACCESS

Continued

REAGENT or RESOURCE	SOURCE	IDENTIFIER
HeLa FIS1 KO clone #13	This study	N/A
HeLa FIS1 KO clone #10	This study	N/A
HeLa TBC1D15 KO clone #5	This study	N/A
HeLa TBC1D15 KO clone #8	This study	N/A
HeLa TBC1D17 KO	This study	N/A
HeLa FIS1/TBC1D15 DKO	This study	N/A
HeLa Drp1 KO	Mariusz Karbowski[52,53]	N/A
HCT116 FIS1 KO	Koji Yamano[7]	N/A
HTC116 WT	ATCC	CVCL_0291
Oligonucleotides		
Forward primer for Gibson assembly of *FIS1* into pBABE-puro	CCTTCTCTAGGCGCCGGCCGGATCCCG CCACCATGGAGGCCGTGCTGAACGAG	N/A
Reverse primer for Gibson assembly of *FIS1* into pBABE-puro	CTAACTGACACACATTCCACAGGGTCG ACTCAGGATTTGGACTTGGACACAGC	N/A
Reverse primer for Gibson assembly of $FIS1^{DC\ (D121\text{-}152)}$ into pBABE-puro	CTAACTGACACACATTCCACAGGGTCGA CTTAATCTTTCTTCATGGCCTTGTCAATG	N/A
Forward primer for Gibson assembly of $FIS1^{DC}$ fragment with *OMP25* fragment (aa 118–145) ($FIS1^{DC\text{-}OMP25}$ chimera)	CATGAAGAAAGATATTCCCATATTTATG GTGCTG	N/A
Reverse primer for Gibson assembly of $FIS1^{DC}$ with OMP25 fragment (aa 118–145) ($FIS1^{DC\text{-}OMP25}$ chimera)	ATATGGGAATATCTTTCTTCATGGCCTT GTCAATGAGC	N/A
Reverse primer for Gibson assembly of OMP25 fragment (aa 118–145) into pBABE-puro ($FIS1^{DC\text{-}OMP25}$ chimera)	CTAACTGACACACATTCCACAGGGTCGA CTCAAAGTTGTTGCCGGTATC	N/A
Forward primer for Gibson assembly of *TBC1D15* into pHAGE-CMV-N-EGFP	GTACAAGTCCGGACTCAGATCTCGAGC TATGGCGGCGGCGG	N/A
Reverse primer for Gibson assembly of *TBC1D15* into pHAGE-CMV-N-EGFP	ATGATCAATCTAGACCGGTGGATCCCTC ATGCAGGTGTTAATCTGC	N/A
FIS1 exon 2 CRISPR forward primer	GTGGGCTGTTGAGCAGTTCT	N/A
FIS1 exon 2 CRISPR reverse primer	TGGGCCTAGGTCTCACAAAAC	N/A
Sequencing primer to verify *FIS1* CRISPR, forward	CACCGTACTCAAACTGCGTGCTCT	N/A
Sequencing primer to verify *FIS1* CRISPR, reverse	AAACAGAGCACGCAGTTTGAGTAC	N/A
TBC1D15 exon 4 CRISPR forward primer	CACCGCCAAAAGAAAGAGGTCATCG	N/A
TBC1D15 exon 4 CRISPR reverse primer	AAACCGATGACCTCTTTCTTTTGGC	N/A
Sequencing primer to verify *TBC1D15* CRISPR, forward	AGCCACCATTGGGCTCTTTT	N/A
Sequencing primer to verify *TBC1D15* CRISPR, reverse	TGTGCATGAAGGACAGCCAAAGG	N/A
TBC1D17 exon 4 CRISPR forward primer	CACCGTCCTCTCCTAAGGACTCCAG	N/A
TBC1D17 exon 4 CRISPR reverse primer	AAACCTGGAGTCCTTAGGAGAGGAC	N/A
Sequencing primer to verify *TBC1D17* CRISPR, forward	GTGTCCATTGAGCACTCCG	N/A
Sequencing primer to verify *TBC1D17* CRISPR, reverse	AGGTTCATAGCCGGGGTCAA	N/A
qPCR *IFNB1*, forward	CTTGGATTCCTACAAAGAAGCAGC	N/A
qPCR *IFNB1*, reverse	TCCTCCTTCTGGAACTGCTGCA	N/A
qPCR *IFIT3*, forward	CGGAACAGCAGAGACACAGA	N/A

(*Continued on next page*)

Continued		
REAGENT or RESOURCE	SOURCE	IDENTIFIER
qPCR *IFIT3*, reverse	CGGAACAGCAGAGACACAGA	N/A
qPCR *ISG15*, forward	TGAGAGGCAGCGAACTCATC	N/A
qPCR *ISG15*, reverse	CAGCATCTTCACCGTCAGGT	N/A
Recombinant DNA		
pLenti-GIII-CMV-TBC1D15	Applied Biological Materials	46231061
pHAGE-CMV-N-mEGFP-TBC1D15	This study	N/A
pBABE-puro-FIS1	This study	N/A
pBABE-puro-FIS1^{DC}	This study	N/A
pBABE-puro-FIS1$^{DC-OMP25}$	This study	N/A
pBABE-puro/3FLAG-TEV-hFIS1	Koji Yamano[15]	N/A
pBABE-puro-HA-Rab7	Koji Yamano[15]	N/A
pBABE-puro-HA-Rab7 QL	Koji Yamano[15]	N/A
pBABE-puro-HA-Rab7 DN	Koji Yamano[15]	N/A
pcDNA3.1-OMP25	Mariusz Karbowski[54]	N/A
pBMN-mCherry-Parkin	Koji Yamano[15]	N/A
pHAGE- CMV-N-mScarlet-STING	Tara D. Fischer (unpublished)	N/A
pHAGE- CMV-N-mEGFP-STING	Tara D. Fischer[55]	N/A
pHAGE- CMV-N-mCherry-Rab7A	Shireen A Sarraf (unpublished)	N/A
pSpCas9(BB)-2A-Puro (PX459) V2.0	Adgene[56]	Addgene plasmid #62988
Software and algorithms		
Fiji	Schindelin et al.[57]	https://imagej.net/software/fiji/
Image Lab	Bio-Rad	https://www.bio-rad.com/en-us/product/image-lab-software?ID=KRE6P5E8Z
SnapGene	SnapGene	https://www.snapgene.com/
Prism	GraphPad	https://www.graphpad.com/features
ZEN	Zeiss	https://www.zeiss.com/microscopy/us/products/software/zeiss-zen.html
Other		
SurePAGE, Bis-Tris, 10 × 8, 4–12%, 10 wells	GenScript	M00652
SurePAGE, Bis-Tris, 10 × 8, 4–12%, 12 wells	GenScript	M00653
Bolt™ Bis-Tris Plus Mini Protein Gels, 8%, 1.0 mm, WedgeWell format	Thermo Fisher Scientific	NW00085BOX
8 well chambered cover glass with #1.5 high performance cover glass	Cellvis	C8-1.5H-N
Aclar 33C Film	Electron Microscopy Sciences	50425
Immobilon® -P PVDF Membrane (0.45 μm pore)	Millipore Sigma	IPVH00010
Slot grids with thick formvar coating	Electron Microscopy Sciences	N/A
Gelatine capsule, size “0”	Electron Microscopy Sciences	N/A
Pelco EasiGlow Glow Discharge Cleaning System	Ted Pella	N/A
Diamond knife	DiATOME, Hatfield, PA, USA	Ultra 45
Leica EM UC7 ultramicrotome	Leica	N/A
Transmission Electron Microscope, 120KV	JEOL USA, Inc., Peabody, MA, USA	JEOL1400 Flash
CMOS detector, 29 Mpix (for electron microscope)	Advanced Microscopy Techniques, Danvers, MA, USA	Biosprint29

(*Continued on next page*)

Continued

REAGENT or RESOURCE	SOURCE	IDENTIFIER
Zeiss LSM 880 Airyscan Confocal Microscope with a 63 × 1.4 NA Plan-Apochromat objective, laser lines (in nm): 405, 488, 561 and 633, Piezo high-precision stage.	Carl Zeiss Microscopy).	N/A
Millex PVDF syringe filter	Millipore Sigma	SLHVR33RB
CFX384 real-time system/C100 Touch Thermal Cycler	Bio-Rad	N/A

EXPERIMENTAL MODEL AND STUDY PARTICIPANT DETAILS

Human and bacterial cell culture

HCT116, HEK 293T and HeLa cells were purchased from ATCC. Cells were cultured in Dulbecco's modified eagle medium with or without phenol red (DMEM, Thermo Fisher Scientific) with 10% (v/v) Fetal Bovine Serum (Sigma), 1 mM Sodium Pyruvate (Thermo Fisher Scientific), and 2 mM GlutaMAX (Thermo Fisher Scientific). Cell lines were stored in Bambanker freezing media (Fujifilm Wako Chemicals). Cells were routinely tested for mycoplasma contamination and contamination was never detected in the cells used for this study. Cell line authentication was not performed.

NEB 5-alpha Competent *E. coli* (New England Biolabs) and NEB Stable Competent *E. coli*, High Efficiency cells (New England Biolabs) were grown on Luria Bertani broth or agar plates (KD Medical). Plasmid-bearing bacteria selection was performed using carbenicillin 100 μg/mL (GoldBio) or kanamycin 50 μg/mL (Sigma-Aldrich).

METHOD DETAILS

Generation of CRISPR gene knockout cell lines

CRISPR sgRNAs targeting common exons by all splice variants of *FIS1*, *TBC1D15*, *TBC1D17* were designed. Oligonucleotides coding for the sgRNA were aligned and cloned into the BbsI-HF site of pSpCas9(BB)-2A-Puro (PX459) V2.0 (Addgene plasmid #62988). HeLa KO cell lines were generated by transfecting cells with the sgRNA plasmid using jetOPTIMUS DNA Transfection Reagent (Polyplus) followed by selection with 1μg/mL of puromycin (Invivogen) for 48h. The transfected cells pool was transferred to media without antibiotic until clonal growth was detected, then single cell colonies were isolated by limiting dilution and expanded in 96-well plates. Screening of KO clones was performed by sequencing the PCR product obtained from the amplified target locus to identify indel generation. Indels were further ratified from clones lacking protein expression of the targeted gene by cloning in pGEM-T Easy (Promega) the PCR product from the amplified sgRNA-targeted locus and then sequencing the cloned inserts. Primers used for sgRNA generation and for KO validation are compiled in the Key resources. HeLa $DRP1^{ko}$ was kindly provided by Dr. Mariusz Karbowski.

Cloning and generation of stably cell line generation

For lentiviral and retroviral vector constructs, inserts were amplified by PCR using Phusion Hot Start II High-Fidelity polymerase (Thermo Fisher) and were cloned by Gibson assembly (New England Biolabs) in the previously digested target vectors (pBABE-puro, pHAGE). TBC1D15 was amplified from pLenti-GIII-CMV-TBC1D15 (Applied Biological Materials), FIS1 from pBABE-puro/3FLAG-TEV-hFIS1, and OMP25 fragment (aa 118–145)[70] from pcDNA3.1-OMP25. Constructs were transformed into NEB 5-alpha Competent *E. coli* or NEB Stable Competent *E. coli*. See key resources table for the complete list of primers used for cloning. pHAGE-mScarlet-STING and pHAGE-mEGFP-STING constructs were kindly donated by Dr. Tara Fischer. pHAGE-mCherry-Rab7A construct was kindly provided by Dr. Shireen Saraf.

HeLa cell lines with stable overexpression of the indicated genes were generated by infection with lentiviruses or retroviruses. Viruses were packaged in HEK293T cells by transfecting the vector and appropriate helper plasmids with polyethylenimine (PEI, Polysciences) prepared in Opti-MEM (Thermo Fisher). Fresh media was added 24h after transfection. Virus-containing media was harvested 24h after the media change, filtered through a 0.45 mm PVDF filter (Millipore Sigma) and added to target HeLa cells with 8 μg/mL polybrene (Sigma-Aldrich). Media was then changed after a 24h incubation with viral-containing media and 2 μg/mL of puromycin (Invivogen) was added to select cells transduced with pBABE-puro derived constructs. Cells were then directly used in experiments or optimized for expression by FACS.

Cell treatments

Cells were plated 48h prior to experiments in 10% (v/v) FBS supplemented DMEM at a density of $-0.1 \times 10^{\hat{}6}$ cells/mL. For treatments with Oligomycin (Calbiochem, Sigma-Aldrich) and Antimycin A (Sigma-Aldrich) were performed as previously reported.[71] To activate cGAS with dsDNA, cells were transfected 4 μg/mL of Poly-dAdT (Invivogen) with lipofectamine 2000 (Thermo Fisher

Scientific) using the standard company protocol. To activate STING, cells were shifted to DMEM containing 1% (v/v) FBS (Thermo Fisher Scientific) 24h prior to the experiment, after washing twice with HBSS (Thermo Fisher Scientific) similarly to how was previously reported.[72] Cells were incubated with media containing H_2O-diluted cGAMP at a final concentration of 15 μg/mL for 8h. *TBC1D15*$^{ko\#5}$+pHAGE-mEGFP or pHAGE-mEGFP-TBC1D15 experiments were performed using 60 mg/mL of cGAMP for 4h. For microscopy experiments, DMEM without phenol red, supplemented with HEPES (Thermo Fisher Scientific), was used. 300 μg/mL of cGAMP for the indicated times was used in experiments where mEGFP or mScarlet-tagged STING was overexpressed. Chloroquine (Sigma-Aldrich) was used to a final concentration of 20mM.

2′3′-cGAMP ELISA

cGAMP production was measured via 2'3'-cGAMP ELISA Kit (Cayman Chemical Company) using the standard protocol provided by the manufacturer. After the specified treatments, –3x10 $^{-6}$ cells were lysed in a cold shaker with M-PER buffer (Thermo Fisher). Protein concentration of clarified lysates was assayed using Pierce BCA Protein Assay (Thermo Fisher Scientific) and ≃20 μg of protein was loaded per well.

Immunoblot assays

After the indicated treatments, cells were washed twice with cold HBSS or 1× PBS (Lonza) and lysed with 1× LDS supplemented with sodium fluoride (Sigma-Aldrich) and cOmplete, Mini, EDTA-free Protease Inhibitor Cocktail (Roche). LDS lysis Buffer was home-made without colorants following the recipe of Thermo Fisher for 4× LDS NuPage buffer (106 mM TRIS-HCl (IBI Scientific), 141 mM Trizma base (Sigma-Aldrich), 2% LDS (Research Products International), 10% glycerol (Bio Basic), 0.51 mM EDTA (Quality Biological)). Samples were lysed by incubating at 99°C, for 10 min shaking at 1000 rpm. Protein concentrations were determined by Pierce BCA Protein Assay, then 20 to 40 μg of DTT-denaturalized (Sigma-Aldrich) protein was separated through cold SDS-PAGE using 4–12% Bis-Tris 1.5 mm precast gels (GenScript) or 8% Bis-Tris 1.0 mm precast gels (Thermo Fisher Scientific). MOPS or MES buffer (GenScript) was used depending on the molecular weight of the proteins to be detected. Gels were electrophoresed at 50V for 20min and then at 100V until the dye front reached the end of the gel and then were transferred to polyvinyl difluoride membranes (0.2 μm (Bio-Rad) or 0.45 μm (Millipore Sigma)) using the Trans-Blot Turbo system (Bio-Rad).

Tris-Buffered Saline containing 0.05% Tween 20 (Sigma-Aldrich) was used to prepare blocking and antibody incubation solutions and to perform membrane washes. For membrane blocking 4% skim milk (Millipore Sigma) was used, and for the antibody incubation, 1, 2 or 4% of BSA (Fisher Scientific) with 0.1% sodium azide (Sigma-Aldrich) was used. Membranes were incubated with primary antibodies either for 2h at RT or O/N at 4°C. The following antibody dilutions were used: pSTING: 1:500 (Cell Signaling Technology); STING: 1:1000 (Cell Signaling Technology), pTBK1: 1:1000 (Cell Signaling Technology), TBK1: 1:1000 (Cell Signaling Technology), pIRF3: 1:1000 (Abcam), IRF3: 1:1000 (Cell Signaling Technology), Fis1: 1:2000 (Proteintech); Actin: 1: 5000 (Sigma-Aldrich); HSP90: 1:5000 (Cell Signaling Technology), TBC1D15: 1:2000 (Abcam), TBC1D17: 1:4000 (Proteintech), HA 1:2000 (Cell Signaling Technology), Rab7A: 1:1000 (NewEast Biosciences), pRab7A: 1:1000 (Abcam), and PINK1: 1:1000 (Cell Signaling Technology). PINK1 antibody was added after blocking with 2% BSA.

Membranes were washed thoroughly and then incubated at RT for 1 or 2h with HRP-linked secondary antibody against the appropriate reactive species, prepared in blocking buffer. After washing secondary antibodies, the membranes were developed using either Amersham ECL Prime Western Blotting Detection Reagent (Cytiva) or SuperSignal West FEMTO Maximum Sensitivity Substrate (Thermo Fisher Scientific) and imaged using a ChemiDoc Imaging System (Bio-Rad). For stripping and re-probing, membranes were incubated with Restore PLUS Western Blot Stripping Buffer (Thermo Fisher) for 1h at RT with shaking.

Immunoprecipitation experiments

For HA-beads (Pierce), specified HeLa cell lines were seeded as previously described and 8–10 × 10^6 cells were lysed at 4°C shaking at 1000 rpm for 30 min in 1× Lysis/Assay Buffer (NewEast Biosciences) supplemented with 2× EDTA-free cOmplete protease inhibitor and 20mM of NaF. Protein concentration of clarified lysates was determined by Pierce BCA Protein Assay and 500 μg of protein was diluted (maintaining 1 part of Assay Buffer to 5 parts of TBSE buffer (20mM Tris-HCl pH 7.6 (Quality Biological), 150mM NaCl (Quality Biological), 1mM EDTA)) and incubated for 2h at 4°C with pre-equilibrated magnetic HA-beads. Then beads were washed at RT 3× with TBSE containing 0.05% Tween 20, 2× cOmplete protease inhibitor and 20mM of NaF. After a wash with ultrapure H_2O, protein was eluted from HA-beads for 10min at 98°C in 30mL of home-made 2× LDS. The eluted fraction was analyzed by immunoblot in parallel to 5% of the input protein lysate (25mg).

The protocol for adherent cells provided by the company with the Rab7 Activation Assay Kit (NewEast Biosciences) was used to prepare the *in vitro* GTPgS/GDP protein loading for positive and negative controls and to immunoprecipitate Rab7A-GTP with anti-Rab7-GTP monoclonal antibody. Briefly, 8–10 × 10^6 cells were lysed as previously described for HA-beads immunoprecipitation, and 800 μg of protein was incubated with 1mL of anti GTP-Rab7and 20 μL of Protein A/G agarose for 2h at 4°C. Beads were then washed 5× with 1× Lysis/Assay Buffer and the protein bound to beads was eluted for 10min at 98°C in 20mL of home-made 2× LDS. The eluted fraction was analyzed by immunoblot in parallel to 2.5% of the input protein lysate (20 μg).

Immunofluorescence microscopy

As described in the previous sections, cells were seeded on #1.5 glass 8 well chambered plates (Cellvis). After the specified treatments, cells were fixed for 15 min at 37°C with either 2 or 4% paraformaldehyde (PFA, Electron Microscopy Sciences) prepared in 1× PBS.

For immunofluorescence detection, cells were then permeabilized with 0.1% or 0.2% Triton X-100 (Sigma-Aldrich). PFA and Triton X- concentrations were adjusted depending on the primary antibodies used. Fixed cells were then incubated with primary antibodies diluted in 1× PBS containing 5% (v/v) FBS (R&D Systems) or 1%BSA (for LAMP1), after being blocked with 10% (v/v) FBS in 1× PBS. The following primary antibodies were used at the specified concentrations for either 2h at RT or O/N at 4°C: TOMM20: 1:1000 (Santa Cruz Biotechnology); FIS1: 1:500; PDI: 1:400 (Thermo Fisher); GM130: 1:200 (BD Transduction Laboratories), TGN46: 1: 500 (Biorad), LAMP1 1:200 (CS). Cells were washed with 5% (v/v) FBS in 1× PBS thoroughly and were then incubated with Alexa Fluor labeled secondary antibodies for 2h at RT or with GFP booster Atto 488 (Proteintech) in 0.2%Triton, 10%FBS in PBS. Secondary antibodies were washed with 5% (v/v) FBS in PBS, and PBS. For nuclei staining, 1:5000 DAPI was used (Thermo Fisher). Samples were conserved in Ibidi mounting medium (Ibidi) and imaged.

Acquisition of images was performed using a Zeiss LSM 880 Airyscan Confocal Microscope with a 63 × 1.4 NA Plan-Apochromat objective, laser lines (in nm): 405, 488, 561 and 633, Piezo high-precision stage and using the Zen software platform (Carl Zeiss Microscopy). The "4 Color FrameShift" configuration of beam splitters and emission filters suggested by Wu and Hammer 2021[73] was used. Zen software was used for Airy-scan image pixel reassignment and deconvolution (using "Auto" setting) for the stitching of tiles. FIJI (ImageJ) was used for merging and aligning channels (to avoid bleaching, TGN channel was acquired independently of other channels in some experiments), to adjust brightness and contrast of the representative images presented, and to prepare images for publication.[74] Some of the microscopy images shown in the Figures were processed with the "smooth" function to ease visualization.

Transmission electron microscopy

Cells stably expressing mEGFP-STING were seeded 48h before being treated on Aclar film coverslips (Electron Microscopy Sciences (EMS)) in 12-well plates. Coverslips were prepared by cutting Aclar into squares that fit into the wells of 12-well plates. Coverslips were previously rinsed in distilled water and sterilized with 70% ethanol and transferred to a 12-well plate, where they were rinsed ten times with autoclaved ultrapure water and rinsed once with culture medium before plating of cells. Cells were left untreated (NT) or were treated with 300μg/mL of cGAMP for 16h and then were fixed at room temperature with 2% paraformaldehyde (EMS), 2% glutaraldehyde (EMS), and 50 mM calcium chloride (Quality Biological, Inc) in 0.1 M sodium cacodylate buffer (EMS). Peroxidase staining was performed with some modifications of the previously described method by Zhang,Q. et al.[75] After fixation, coverslips were rinsed twice in 0.1 M sodium cacodylate buffer, followed by a 10-min incubation in cacodylate buffer containing 50 mM glycine (Sigma-Aldrich) and a final rinse in cacodylate buffer. Coverslips were rinsed twice with cacodylate buffer and then post-fixed with 3% glutaraldehyde in cacodylate buffer. For EM processing, coverslips were rinsed twice for 10 min in cacodylate buffer, and then stained with reduced osmium containing 1% osmium tetroxide (EMS) and 1% potassium ferrocyanide (Sigma-Aldrich) on ice for 60 min. Next, coverslips were rinsed three times with cacodylate buffer and dehydrated through a graded series of 5-min ethanol rinses prior to infiltration in EmBED812 epoxy resin using the manufacturer's hard resin formulation (EMS). To embed cells, Aclar coverslips were laid cell-side-up on a backing piece of Aclar, and then 2–3 gelatine capsules (EMS) filled with resin were inverted on top of the coverslips and placed in a 60°C oven for 2 days.

For sectioning, Aclar coverslips were peeled away from the resin, and *en face* ultrathin sections of embedded cells were cut to a thickness of 70 nm using an ultramicrotome (EM UC7, Leica Microsystems) with a diamond knife (DiATOME). Sections were picked up on 1 mm-slot copper grids with a thick formvar support film (EMS), and post-stained with 3% Reynold's lead citrate (EMS) for 1 min. Sections were viewed using a JEOL1400 Flash transmission electron microscope (JEOL USA, Inc.) operated at 120 KV. Images were recorded with a Biosprint29 CMOS detector (Advanced Microscopy Techniques). FIJI (ImageJ) was used to visualize and to adjust brightness and contrast of the representative images presented, and to prepare images for publication.[74]

Quantitative real-time PCR

Total RNA from 5–10 × 10^5 cells was extracted using Quick-RNA MiniPrep Plus kit (Zymo Research) followed by reverse transcription using High-Capacity cDNA Reverse Transcription Kit (Thermo Fisher Scientific). Equal amounts of cDNA and corresponding primers were used for qPCR using SYBR Green Master Mix (Thermo Fisher Scientific) and a CFX384 real-time system/C100 Touch Thermal Cycler (Bio-Rad).

QUANTIFICATION AND STATISTICAL ANALYSIS

ELISA-detected cGAMP concentrations were calculated using the manufacturer-provided spreadsheet, values were normalized to the calculated protein concentration per well and graphed using Graphpad Prism 10.

The qPCR obtained results, for each biological sample, the Ct value of the gene of interest was normalized against the b-actin Ct to calculate ΔCt. Each ΔCt was normalized to the average ΔCt of untreated samples to generate the ΔΔCt value. Relative gene expression was then analyzed using the 2-ΔΔCt formula.[76] Results were plotted and statistically analyzed using Graphpad Prism 10.

Densitometric quantifications of immunoblot images were performed using the FIJI Gel analysis tool.[74] The pixel values obtained from quantifying three independent blots for pSTING, STING, pTBK1, TBK1, pIRF3, IRF3, pRAB7A and RAB7A were normalized using the internal loading control (HSP90 or Actin) and were plotted either relativized to the WT condition without treatment or as the ratio between cGAMP treated to untreated conditions. The immunoprecipitated TBC1D15 to HA ratio was calculated from the pixels of blot images from 4 co-immunoprecipitation assays.

To calculate STING and TIMM17A half-life, the natural logarithm (ln) of normalized protein levels at each time point relative to t = 0 from three independent experiments was plotted. Then a Simple Linear regression was generated, and the half-life ($t_{1/2}$) was calculated using the equation $N(t) = N_0 e^{-kt}$, in which k is the slope of the obtained line and $t_{1/2} = \ln(2)/k$.

To quantify STING trafficking or TBC1D15 localization at the mitochondria, the percentage of cells in which mEGFP signal colocalized respectively with 633 or 405 channels was quantified manually guided by images generated by the FIJI plugin Colocalization finder. The proportion of mEGFP-STING signal within mCherry labeled organelles or in LAMP1-positive vesicles, and of mEGFP-TBC1D15 signal within Golgi markers or LAMP1-positive vesicles, was performed using FIJI. Same size regions of individual cells (regions of interest: ROIs) were thresholded using respectively the 405, 561 or 633 channels to generate a mask. Then the fluorescence inside the mask, total fluorescence of the ROIs and background were calculated and the proportion inside/outside the mask was calculated and plotted as percentage. Pearson's correlation coefficient was calculated from same size ROIs of individual cells using Coloc 2 FIJI plug in.

Organelles were manually classified based on morphological characteristics in cell ultrastructure images acquired by TEM and the area was manually quantified using FIJI.

Plots and statistical analysis were performed using Graphpad Prism 10 and statistical details can be found in the figure legends. Each point value and all relevant *p*-values obtained from the statistical comparisons performed are depicted in the graphs.

Plots and statistical analysis were performed using Graphpad Prism 10. To analyze the difference between two groups a two-tailed Student's *t* tests was used. To assay multi-group comparisons a one-way ANOVA followed by a post hoc test was performed (Sidak's or Bonferroni). For experiments involving two independent variables, two-way ANOVA followed by Bonferroni pos hoc test was performed. An alpha of 0.1 was set when performing comparisons. Data are expressed as mean ± SD from at least three independent experiments. Specific statistical details and the number of cells analyzed for imaging analyses can be found in the figure legends. Each point value and all relevant *p*-values obtained from the statistical comparisons performed are depicted in the graphs.

Cell Reports, Volume *45*

Supplemental information

Mitochondrial Fis1 tunes Rab7A activity

via TBC1D15 modulating STING degradation

Eunice Dominguez-Martin, Linlin Lei, Jennifer D. Petersen, Peng-Peng Zhu, Katherine R. Tucker, and Richard J. Youle

Figure S1. STING degradation is impaired in the absence of Fis1, related to Figure 1. (A) STING, Fis1 and Hsp90 protein levels detected by immunoblotting whole-cell lysates from WT and $FIS1^{ko\#10}$ cells treated with cGAMP or left untreated. **(B)** Densitometric quantifications of STING levels in experiments as in A (NT= no treatment, AU= Arbitrary units, n=3, mean±SD, paired t test: WT+cGAMP vs $FIS1^{ko\#10}$+cGAMP). **(C)** Immunoblot to detect STING and Fis1 protein levels in whole-cell lysates from WT, $FIS1^{ko\#13}$, WT+pBABEpuro or $FIS1^{ko\#13}$+pBABEpuro, induced with cGAMP or left untreated. Actin was included as a loading control. **(D)** Quantification of STING protein levels of experiments as in C (AU= Arbitrary units, n=3, mean±SD, one-way ANOVA, Bonferroni). **(E)** STING degradation in WT and $FIS1^{ko\#}$ HCT116 cells treated with cGAMP or left untreated. Actin was used as loading control and Fis1 absence was corroborated. **(F)** STING protein levels as a ratio of cGAMP/NT of experiments as in (E) (n=3, mean±SD, two-tailed paired *t-test*). **(G)** Mitophagy induction determined by PINK1 stabilization and detected by immunoblotting whole cell lysates of WT HeLa cells stably expressing mCherry-Parkin (mCh-PRKN) after the specified treatments. STING was also blotted, and Actin was included as a loading control. **(H)** Immunoblot assaying STING levels in WT, $FIS1^{ko\#13}$, $FIS1^{ko\#13}$+$Fis1^{\Delta C}$ and $FIS1^{ko\#13}$+$Fis1^{WT}$ cells stimulated with cGAMP or with no treatment (NT). Fis1 expression was verified, and Actin was blotted as a loading control. **(I)** STING protein levels quantification as a ratio of cGAMP/NT of experiments in (H) (NT= no treatment, AU= Arbitrary units, n=3, mean±SD, one-way ANOVA, Bonferroni). **(J)** STING protein levels assayed by immunoblot in $FIS1^{ko\#13}$ cells stably expressing either $Fis1^{WT}$, a cytosolic C-term truncation: $Fis1^{\Delta C}$, or the $Fis1^{\Delta C\text{-}OMP25}$ mitochondria-anchored chimera. Fis1 was also detected, and Actin was blotted as a loading control. **(K)** Densitometries of STING protein levels as a ratio of cGAMP/NT of experiments as in (H) (n=3, mean±SD, one-way ANOVA, Bonferroni). **(L)** Confocal images of $FIS1^{ko\#13}$ cells expressing either $Fis1^{WT}$, $Fis1^{\Delta C}$, or $Fis1^{\Delta C\text{-}OMP25}$ processed for the detection of Fis1 and TOMM20 by immunofluorescence. The Pearson's correlation of Fis1 vs TOMM20 was quantified. Scale bar = 10 µm. **(M-N)** Plots of the natural logarithm (ln) of STING **(M)** or TIMM17A **(N)** normalized protein levels relative to t=0h from 3 experiments as in Figure 1D. A linear regression was obtained from the data plotted in Figure 1 and the respective line equations are displayed.

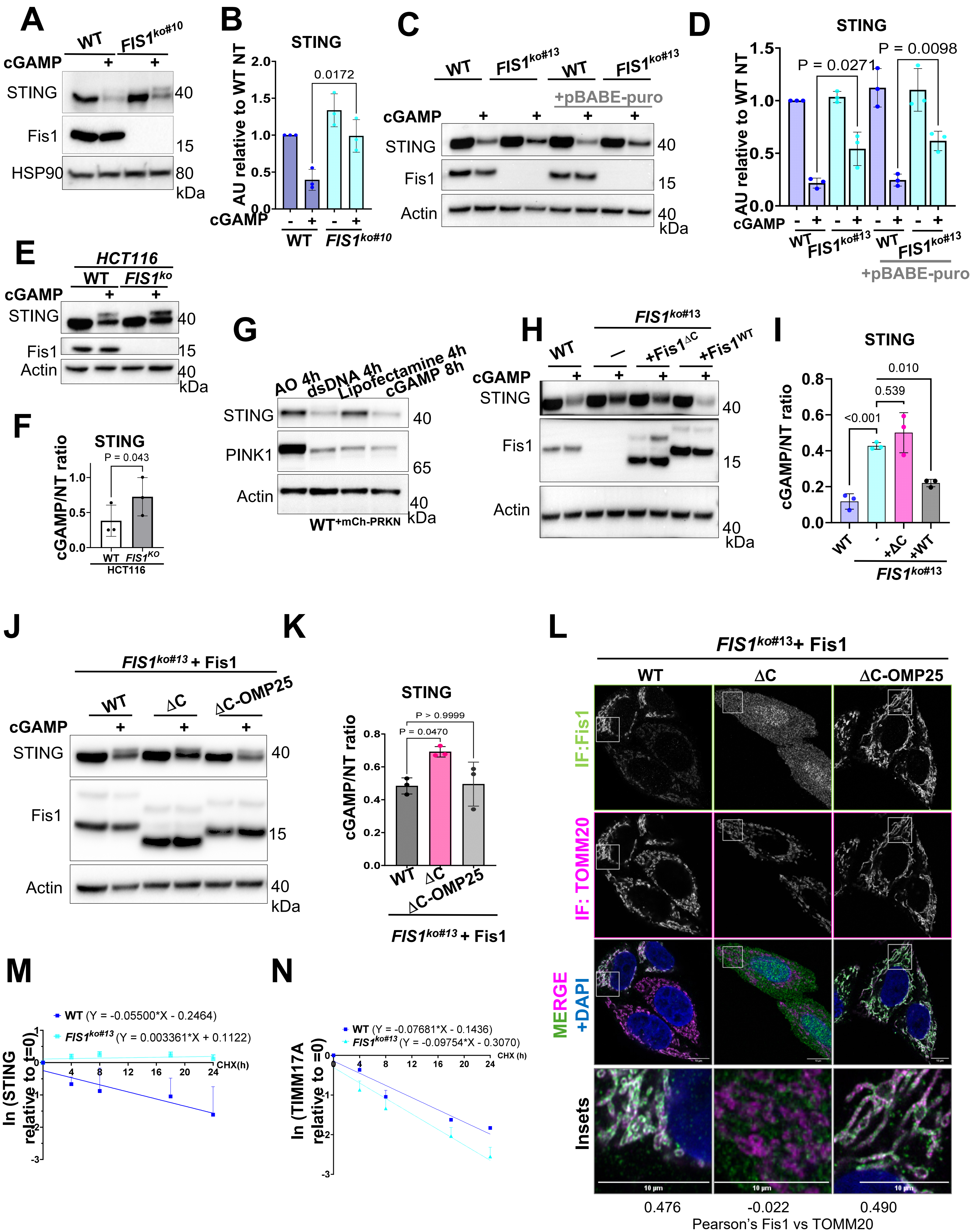
A
WT
FIS1ko#10
cGAMP
+
STING
40
Fis1
15
HSP90
80
kDa
B
STING
AU relative to WT NT
0.0172
cGAMP
WT
FIS1ko#10
C
WT
FIS1ko#13
+pBABE-puro
cGAMP
STING
Fis1
Actin
40
15
40
kDa
D
STING
P = 0.0271
P = 0.0098
AU relative to WT NT
cGAMP
WT
FIS1ko#13
+pBABE-puro
E
HCT116
WT
FIS1ko
cGAMP
STING
Fis1
Actin
40
15
40
kDa
F
STING
P = 0.043
cGAMP/NT ratio
WT
FIS1KO
HCT116
G
AO 4h
dsDNA 4h
Lipofectamine 4h
cGAMP 8h
STING
PINK1
Actin
40
65
40
kDa
WT+mCh-PRKN
H
FIS1ko#13
WT
+Fis1ΔC
+Fis1WT
cGAMP
STING
Fis1
Actin
40
15
40
kDa
I
STING
cGAMP/NT ratio
<0.001
0.539
0.010
WT
-
+ΔC
+WT
FIS1ko#13
J
FIS1ko#13 + Fis1
WT
ΔC
ΔC-OMP25
cGAMP
STING
Fis1
Actin
40
15
40
kDa
K
STING
P > 0.9999
P = 0.0470
cGAMP/NT ratio
WT
ΔC
ΔC-OMP25
FIS1ko#13 + Fis1
L
FIS1ko#13+ Fis1
WT
ΔC
ΔC-OMP25
IF:Fis1
IF: TOMM20
MERGE
+DAPI
Insets
10 μm
0.476
-0.022
0.490
Pearson's Fis1 vs TOMM20
M
WT (Y = -0.05500*X - 0.2464)
FIS1ko#13 (Y = 0.003361*X + 0.1122)
ln (STING relative to t=0)
CHX(h)
N
WT (Y = -0.07681*X - 0.1436)
FIS1ko#13 (Y = -0.09754*X - 0.3070)
ln (TIMM17A relative to =0)
CHX (h)

Figure S2. STING trafficking in WT and *FIS1*^{ko} HeLa cells, related to Figure 2.

Confocal microscopy analysis of STING subcellular localization in WT and *FIS1*$^{ko\#13}$ HeLa cells expressing mEGFP-STING. Cells were treated with cGAMP for the indicated times (h) and stained to detect by immunofluorescence the indicated marker proteins: PDI (Endoplasmic Reticulum), TGN46 (Trans Golgi Network). Scale bars: 10µm.

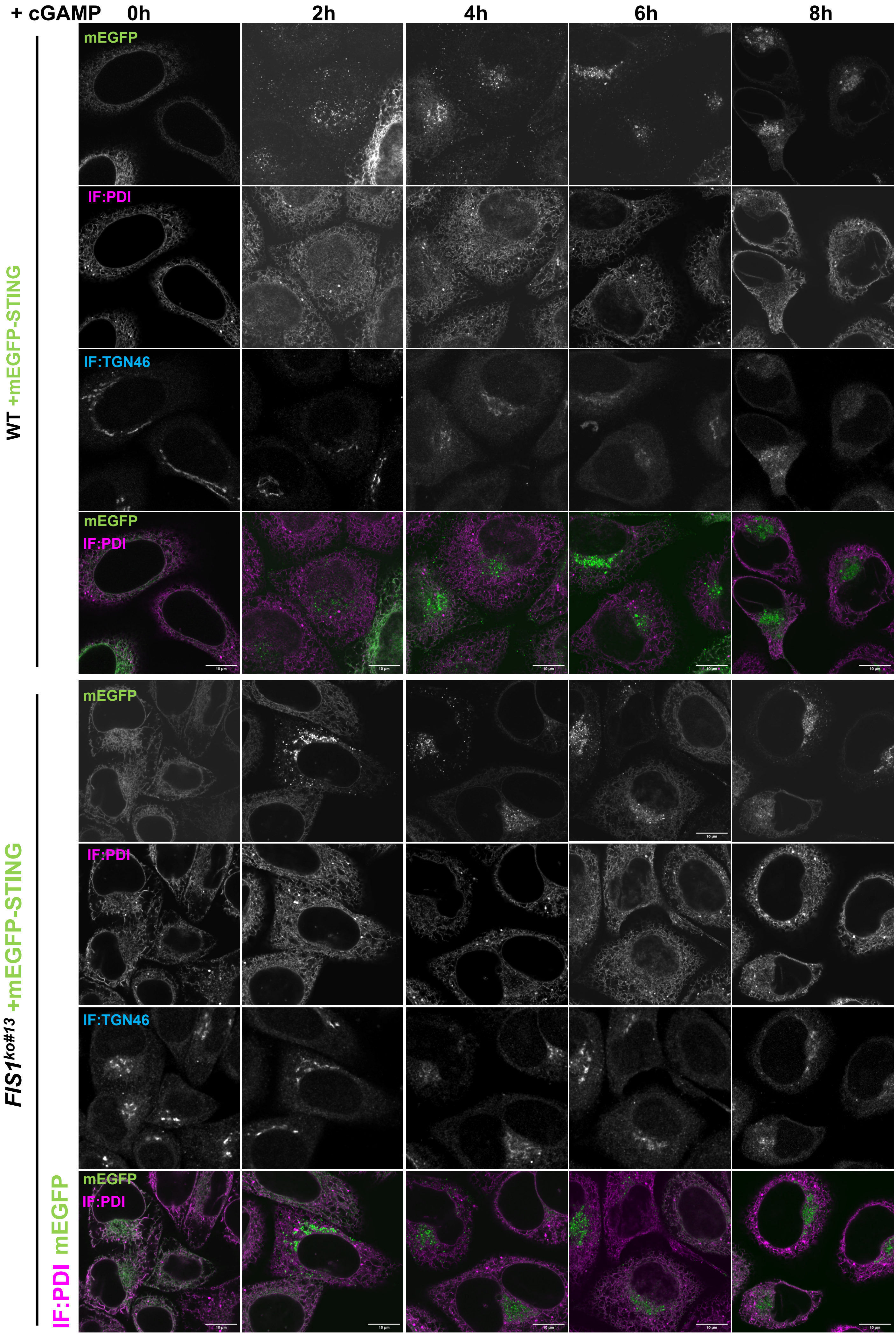

+ cGAMP
0h
2h
4h
6h
8h
WT +mEGFP-STING
mEGFP
IF:PDI
IF:TGN46
mEGFP
IF:PDI
FIS1ko#13 +mEGFP-STING
mEGFP
IF:PDI
IF:TGN46
mEGFP
IF:PDI
IF:PDI mEGFP

Figure S3. Rab7A localization during STING trafficking, related to Figure 3.
(A) Confocal microscopy time-course imaging of mCherry-Rab7 localization in WT or $FIS1^{ko\#13}$ cells expressing mEGFP-STING, treated with cGAMP for the indicated times (h) and stained to detect by immunofluorescence TGN46 (Trans Golgi Network 46). Scale bars: 10µm. **(B)** Immunoblot analysis of STING in WT and $RAB5^{kd}$ cells upon a cGAMP treatment. Rab5 expression was assessed and HSP90 was used as loading control. **(C)** Densitometric quantification of immunoblot signals from (B) (n=3, mean ±SD, paired two-tailed *t-test*: WT vs $RAB5^{kd}$). **(D)** STING protein levels in WT and $RAB11^{kd}$ cells upon a cGAMP treatment. Rab11 expression was assessed and HSP90 was used as loading control. **(C)** Densitometric quantification of immunoblot signals from (D) (n=3, mean ±SD, paired two-tailed *t-test*: WT vs $RAB11^{kd}$).

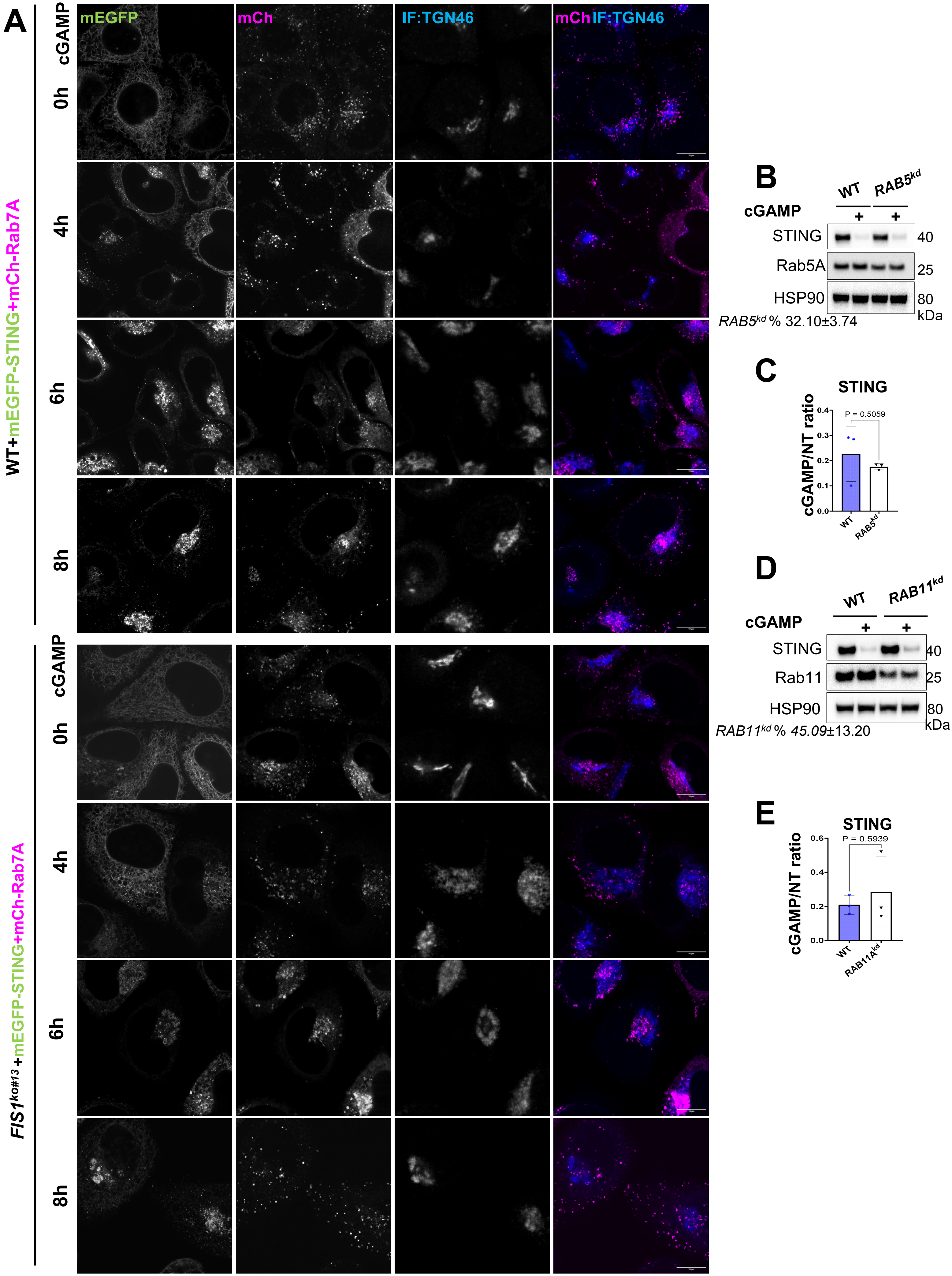
A
mEGFP
mCh
IF:TGN46
mCh IF:TGN46
cGAMP
0h
4h
6h
8h
WT+mEGFP-STING+mCh-Rab7A
FIS1ko#13+mEGFP-STING+mCh-Rab7A
B
WT
RAB5kd
cGAMP
+
+
STING
Rab5A
HSP90
40
25
80
kDa
RAB5kd % 32.10±3.74
C
STING
P = 0.5059
cGAMP/NT ratio
0.0
0.1
0.2
0.3
0.4
WT
RAB5kd
D
WT
RAB11kd
cGAMP
+
+
STING
Rab11
HSP90
40
25
80
kDa
RAB11kd % 45.09±13.20
E
STING
P = 0.5939
cGAMP/NT ratio
0.0
0.2
0.4
0.6
WT
RAB11Akd

Figure S4. The absence of Fis1 or TBC1D15 does not alter mitochondrial morphology and does not induce cGAMP accumulation in HeLa cells, related to Figure 4. (A) STING and TBC1D15 protein levels assayed in WT or *TBC1D15$^{ko\#8}$* cells upon cGAMP stimulation. Actin was blotted as a loading control. **(B)** Densitometries of STING levels in A (NT= no treatment, AU= Arbitrary units,n=3, mean±SD, paired t test: WT+cGAMP vs *TBC1D15$^{ko\#8}$*+cGAMP). **(C)** Immunoblot to detect STING levels in WT and *TBC1D15$^{ko\#5}$* cells with and without the expression of pHAGE-mEGFP upon a cGAMP treatment. **(D)** Quantification of STING protein levels as a ratio of cGAMP/NT of experiments as in C (n=3, mean±SD, one-way ANOVA, Bonferroni). **(E to J)** Representative maximum projections of Airy-scan processed confocal images in which mitochondria were labeled by immunofluorescence against TOMM20 in WT **(E)**, *DRP1^{ko}* **(F)**, *FIS1$^{ko\#13}$* **(G)**, *FIS1$^{ko\#10}$***(H)**, *TBC1D15$^{ko\#5}$* **(I)** or *TBC1D15$^{ko\#8}$* **(J)** cells. Nuclei were stained with DAPI. Insets show magnifications of peripheral mitochondria. Scale bar = 10 µm. **(K)** cGAMP levels (pg/µg of protein) detected by ELISA in whole cell lysates of WT, *FIS1$^{ko\#13}$* or *TBC1D15$^{ko\#5}$* cells stably expressing mCh-PRKN after an Antimycin + Oligomycin (AO) or mock treatment for 4h, a lysate of WT cells transfected with 4 µg/ml of poly-dAdT (dsDNA) was included as a positive control (dsDNA) (one-way ANOVA, Bonferrioni). For all images, *TBC1D15^{ko}=D15^{ko}*.

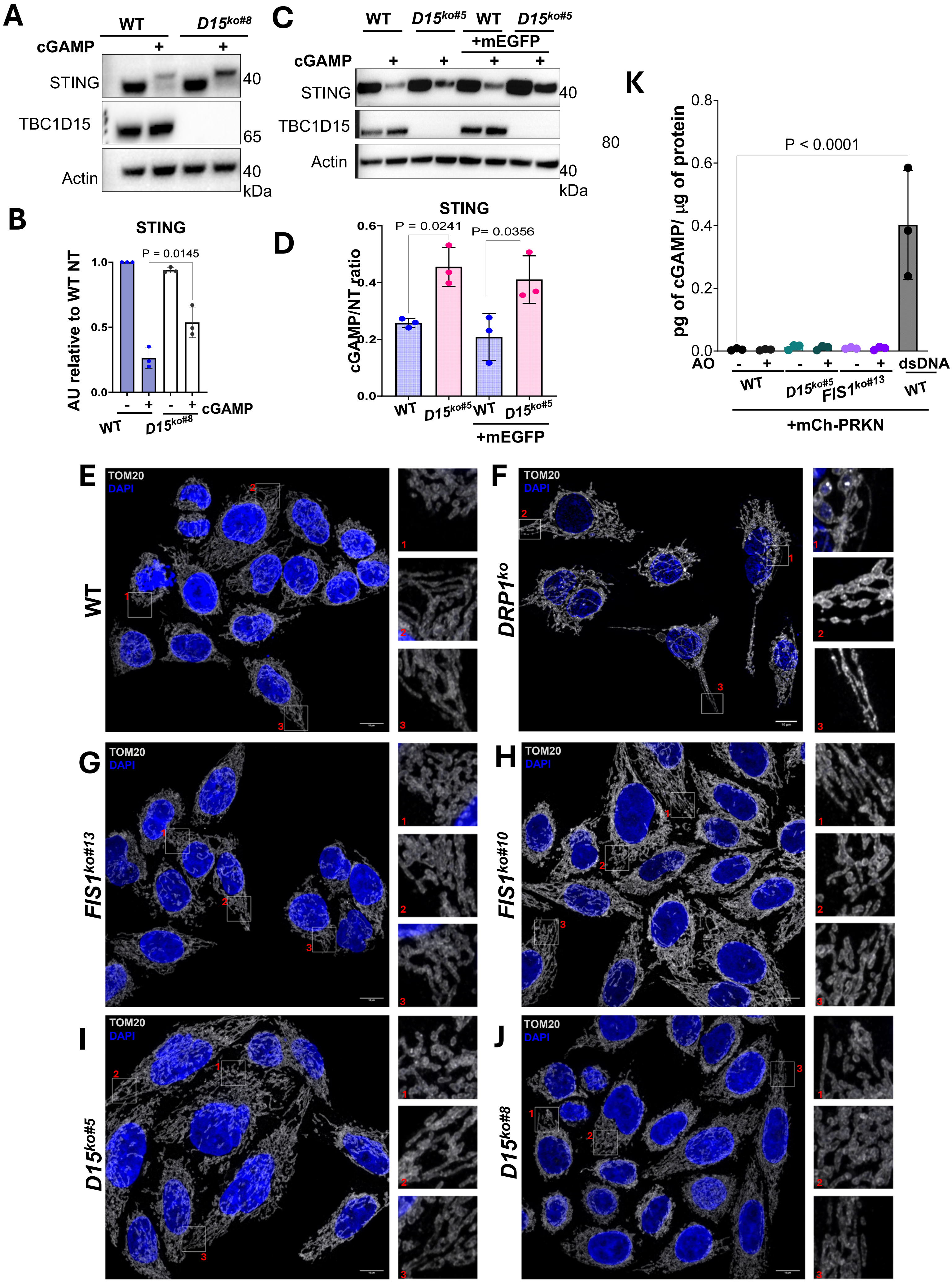

A
WT
D15ko#8
cGAMP
STING
TBC1D15
Actin
40
65
40
kDa
B
STING
AU relative to WT NT
P = 0.0145
cGAMP
WT
D15ko#8
C
WT
D15ko#5
WT
D15ko#5
+mEGFP
cGAMP
STING
TBC1D15
Actin
40
80
40
kDa
D
STING
cGAMP/NT ratio
P = 0.0241
P= 0.0356
WT
D15ko#5
WT
D15ko#5
+mEGFP
K
pg of cGAMP/ μg of protein
P < 0.0001
AO
dsDNA
WT
D15ko#5
FIS1ko#13
WT
+mCh-PRKN
E
TOM20
DAPI
WT
F
DRP1ko
G
FIS1ko#13
H
FIS1ko#10
I
D15ko#5
J
D15ko#8

Figure S5. mEGFP-TBC1D15 subcellular localization is influenced by Fis1 expression levels, relative to Figures 5 and 6. (A) mEGFP-TBC1D15 subcellular localization in *FIS1/TBC1D15*$^{dko\#9}$ cells lacking Fis1 expression (-) or in which either Fis1^{WT} or cytosolic Fis1$^{\Delta C}$ was reintroduced. Fis1 was detected via immunofluorescence. Images are representative stitched Airy-scan processed tiles. **(B)** Pearson's correlation was measured to assess colocalization of mEGFP-TBC1D15 and Fis1 forms in (A) (n=17 and 18 cells, two-tailed unpaired *t-test*). **(C)** Colocalization of mEGFP-TBC1D15 with TOMM20 detected by immunofluorescence acquired by imaging *FIS1/TBC1D15*$^{dko\#9}$ cells stably expressing mSca-STING and both Fis1 and mEGFP-TBC1D15. **(D)** Airy-scan processed images of mEGFP-TBC1D15 expressed in *FIS1/TBC1D15*$^{dko\#9}$+mSca-STING cells. The TGN was detected by staining TGN46 by immunofluorescence. **(E)** Subcellular localization of mCh-TBC1D15 in *FIS1/TBC1D15*$^{dko\#9}$ cells expressing either mEGFP-tagged WT Fis1 or Δ77-98 mutant Fis1. Mitochondria were detected by immunostaining TOMM20 and the TGN by detecting TGN46. **(F)** Pearson's coefficient of mCh-TBC1D15 vs TOMM20 in cells expressing either of WT Fis1 or Δ77-98 mutant Fis1 was quantified to assess mitochondria localization of TBC1D15 (n= 9 and 8, unpaired two-tailed *t-test*) and of **(G)** mCh-TBC1D15 vs TGN46 to assess TBC1D15 localization at Golgi (n= 7 and 10, unpaired two-tailed *t-test*). In all images, the scale bar = 10µm. For all images, *TBC1D15*=*D15*.

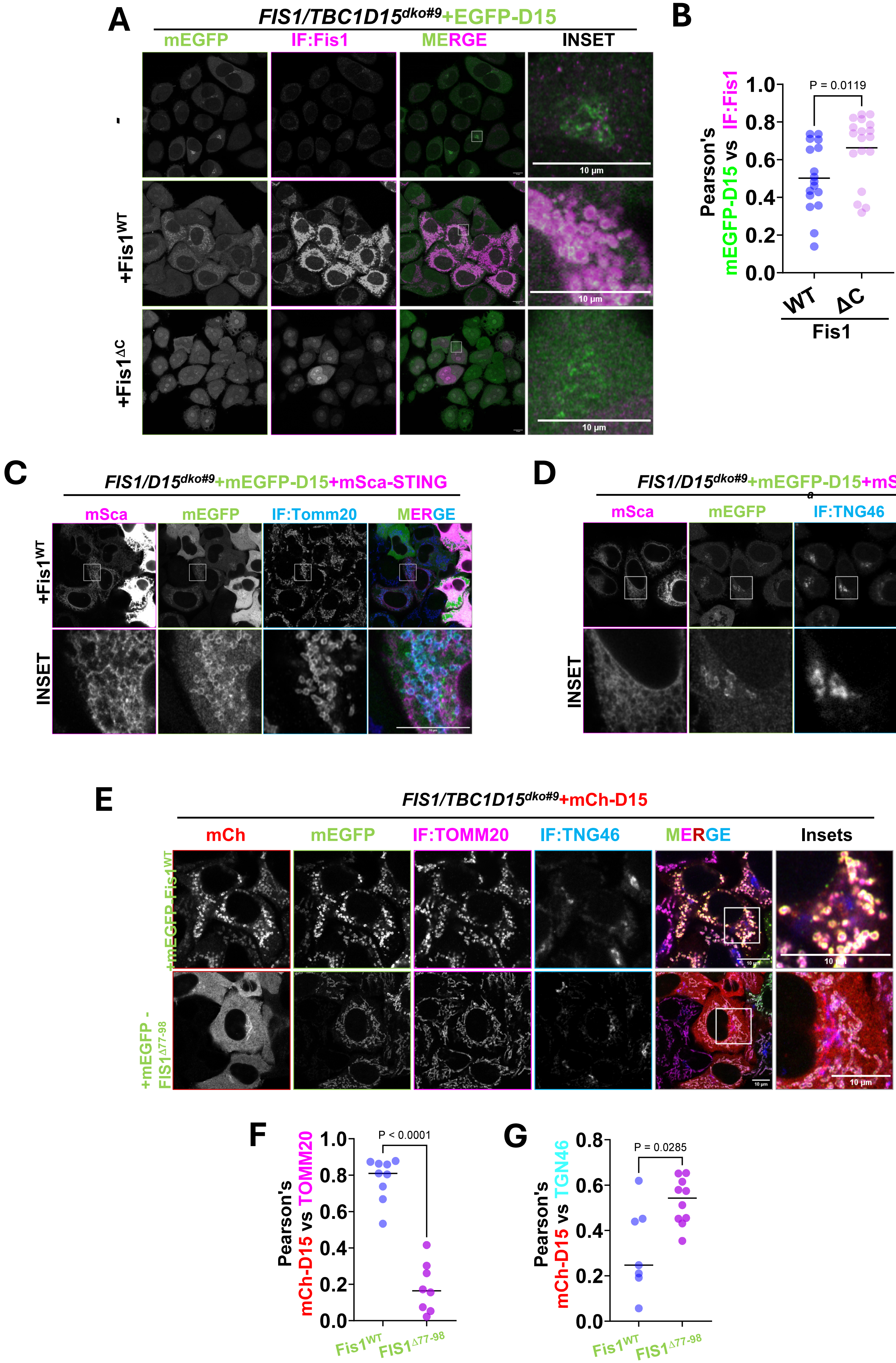
A
FIS1/TBC1D15dko#9+EGFP-D15
mEGFP
IF:Fis1
MERGE
INSET
-
+Fis1WT
+Fis1ΔC
10 µm
B
Pearson's mEGFP-D15 vs IF:Fis1
P = 0.0119
WT
ΔC
Fis1
C
FIS1/D15dko#9+mEGFP-D15+mSca-STING
mSca
mEGFP
IF:Tomm20
MERGE
+Fis1WT
INSET
D
FIS1/D15dko#9+mEGFP-D15+mSca-STING
mSca
mEGFP
IF:TNG46
MERGE
INSET
E
FIS1/TBC1D15dko#9+mCh-D15
mCh
mEGFP
IF:TOMM20
IF:TNG46
MERGE
Insets
+mEGFP-Fis1WT
+mEGFP - FIS1Δ77-98
10 µm
F
Pearson's mCh-D15 vs TOMM20
P < 0.0001
Fis1WT
FIS1Δ77-98
G
Pearson's mCh-D15 vs TGN46
P = 0.0285
Fis1WT
FIS1Δ77-98
